# Effect of dietary peNDF levels on digestibility and rumen fermentation, and microbial community in growing goats

**DOI:** 10.3389/fmicb.2022.950587

**Published:** 2022-08-25

**Authors:** Jia Zhou, Benchu Xue, Anhai Hu, Shuangming Yue, Mei Wu, Qionghua Hong, Yuhan Wu, Zhisheng Wang, Lizhi Wang, Quanhui Peng, Bai Xue

**Affiliations:** ^1^Institute of Animal Nutrition, Sichuan Agricultural University, Chengdu, China; ^2^Department of Bioengineering, Sichuan Water Conservancy College, Chengdu, China; ^3^Yunnan Animal Science and Veterinary Institute, Kunming, China

**Keywords:** goat, peNDF, nutrient digestibility, ruminal fermentation, microbial community

## Abstract

Physically effective neutral detergent fiber (peNDF) is a concept that accounts for the particle length of NDF in diets, sustaining the normal chewing behavior and rumen fermentation of ruminants. Specifically, peNDF_>1.18_ is the commonest one that is calculated from NDF and the percentage of feed dry matter left on the 1.18, 8.00, and 19.00 mm sieves. This study aimed to investigate the effects of different levels of peNDF_>1.18_ on the rumen microbiome and its correlation with nutrient digestibility and rumen fermentation in goats. A total of 30 Lezhi black goats were randomized and blocked to five dietary treatments (*n* = 6). All the diets were identical in composition but varied in hay lengths, leading to the different peNDF_>1.18_ content of the diets: 32.97, 29.93, 28.14, 26.48, and 24.75%. The results revealed that the nutrient digestibility increased when dietary peNDF_>1.18_ levels decreased from 32.97% to 28.14%, with the highest digestibility at 28.14% peNDF_>1.18_ treatment, after which nutrient digestibility decreased with the decreasing of dietary peNDF levels. Ruminal NH_3_-N concentrations in the 29.93% and 28.14% groups were higher than that in the 24.75% group (*p* < 0.05). Ruminal microbial protein concentration was the highest in the 32.97% group (*p* < 0.05). Daily CH_4_ production in the 32.97% and 24.75% peNDF_>1.18_ treatments was lower than that in the 26.48% group (*p* < 0.05) and no differences were observed among other groups. The relative abundance of rumen fungi at the phylum and genus levels and archaea at the species were affected by dietary peNDF_>1.18_ content. In conclusion, decreasing dietary peNDF_>1.18_ levels within a certain range can improve nutrient digestibility and change the rumen microbial community structure of goats. Dietary peNDF_>1.18_ level should be 28.14% (roughage length around 1 cm) among the five levels for 4 months Lezhi black goats with the purpose of optimal nutrient digestibility.

## Introduction

Forages comprise more than 40% of the diets for ruminants and are essential to maintain an appropriate rumen function and physiology (Bargo et al., 2002). The high fiber content is the major nutritional difference between forages and concentrates, resulting in a lower energy value of forages. Unlike monogastric animals, the rumen of ruminants is colonized with an abundance of microorganisms, which are capable of converting fiber in feed into available energy for the host. Meanwhile, fiber can stimulate chewing, salivation, rumination, and ruminal motility of ruminants. It also plays an important role in alleviating rumen acidosis, regulating dietary intake, synthesizing milk fat, and promoting the digestion of solid particles in the rumen (Adesogan et al., 2019). Consequently, evaluating and improving the utilization efficiency of dietary fiber is particularly important for the formulation of diets for ruminants.

In order to assess the adequacy of dietary fiber for ruminants, the concept of physically effective neutral detergent fiber (peNDF) was proposed by Mertens (Mertens, 1997) and attracted more and more attention. The peNDF is more efficient to indicate the physical effects of a diet because it incorporates information on particle size and chemical fiber content of the feedstuffs (Zebeli et al., 2012) and reflects the physical characteristics that influence masticatory activity and stimulate stratification of the rumen (Zhao et al., 2011). It seems to be an ideal and versatile method to calculate the peNDF by separating the diet or forage into pellets of various grain sizes using a manually operated Penn State Particle Separator (PSPS). The peNDF content in feedstuffs or forages could be determined by their neutral detergent fiber (NDF) content of them multiplied by the proportion of pellet retained on the 1.18-mm sieve or 8.00-mm sieve, defined as peNDF_>1.18_ and peNDF_>8.00_, respectively (Zebeli et al., 2012).

The rumen is a very important digestive organ of ruminants, hosting a large number of microorganisms that can effectively degrade proteins and carbohydrates through fermentation. The efficiency of rumen digestion mainly depends on microorganisms that may be affected by dietary peNDF (Li et al., 2014). It was well documented that increasing the dietary peNDF content resulted in increased time for ruminating and chewing (Yang and Beauchemin, 2007; Zhao et al., 2011; Li et al., 2014). However, the effects of dietary peNDF levels on nutrient digestibility are not consistent in the literature. Several recent studies revealed that the increase of dietary peNDF content could reduce the total tract apparent digestibility of dry matter (DM), crude protein (CP), and NDF in dairy cattle (Soita et al., 2002; Molavian et al., 2020) and lambs (de Paula Carlis et al., 2021). Other studies found that the nutrients digestibility was quadratically related to dietary peNDF content (Zhao et al., 2011; Wang et al., 2017), or not affected by peNDF (Oh et al., 2016; Jang et al., 2017; Kaeokliang et al., 2019; Li et al., 2020). In addition, nutrient digestibility is influenced by the rumen microbiome (Li and Guan, 2017). Previous studies have reported that dietary fiber is an important factor influencing rumen microbial composition, diversity, and microbial metabolites (Billenkamp et al., 2021; Yildirim et al., 2021). But till now, few studies have been conducted concerning the effects of dietary peNDF on rumen microbial community structure and the relationship between rumen microbiota and nutrient digestibility, especially fungi.

The unique rumen structure of ruminants allows goats to effectively utilize some unconventional feed resources such as straw and forage. To make more efficient use of feed resources such as straw, it is crucial to evaluate the nutritional value and its effect on various aspects including nutrient digestibility, rumen fermentation, and rumen microbial communities, which could dramatically influence the production performance and health condition of ruminant (Cao et al., 2021), thereby providing useful information and guidance for improving the production efficiency of goats. We hypothesized that variation in the dietary peNDF levels could affect nutrient digestibility, rumen fermentation, and rumen microbial diversity. The objective of this study was to investigate the effects of dietary peNDF levels on nutrient digestibility, rumen fermentation, and rumen microbial communities of Lezhi black goats, by analyzing the predicted functional differences of different ruminal microorganisms.

## Materials and methods

### Experimental design, animals

All experimental protocols were approved by the Animal Ethical and Welfare Committee of Animal Nutrition Institute, Sichuan Agricultural University (Approval No.201408-3).

This study was a part of a larger project which aimed to explore the effect of dietary peNDF level on the development of the digestive tract in growing goats. Animal feeding and management were described previously by Xue et al. (2022). In brief, a total of 30 male Lezhi black goats, aged 4.0 ± 0.1 months and weighed 21.42 ± 0.24 kg, were randomly blocked into five treatments, with six replicates per treatment and one goat per replicate. The diet composition and nutrient levels were the same for the five treatments except for the level of peNDF. The diets were formulated according to the nutrient requirements of 20 kg goats with a daily weight gain of 100 g by NRC (2007), and the ingredients and chemical composition as shown in Table 1.

**Table 1:** Ingredients and chemical composition of experimental diet.

**Items**	**% of dry matter**
Ingredients	
Ground corn	37.10
Soybean meal	2.30
Wheat bran	2.90
Alfalfa hay	10.00
Peanut vine	9.00
Leymus chinensis	36.0
Calcium carbonate	0.17
Calcium hydrogen phosphate	1.02
Sodium chloride	0.51
Premix^a^	1.00
Total	100.00
Chemical composition^b^	
Metabolic energy, ME (MJ/kg)	10.23
Crude protein	8.87
Neutral detergent fiber	42.89
Acid detergent fiber	30.5
Calcium	0.74
Phosphorus	0.87

a Premix (per kg) contains: Cu 1,800 mg, Fe 4,000 mg, Zn 6,500 mg, Mn 8,000 mg, I 100 mg, Se 5 mg, Vitamin A 800 kIU, Vitamin D 120 kIU, Vitamin E 50 kIU, Vitamin K_3_ 200 mg, Vitamin B_1_ 200 mg, Vitamin B_12_ 5 mg.

b Metabolic energy is calculated, and the remaining indicators are measured values.

Dietary peNDF levels were controlled by cutting the forage (*Leymus chinensis*, alfalfa, peanut vine, all of them are hays) at the following lengths with a forage cutter (FS60, Jining Nongfengli Machinery Equipment Co., Ltd., Jining, China): long (7 cm), medium (4 cm), short (1 cm), fine (5 mm sieve), very fine (1 mm sieve). Particle size distribution of forages and mixed diets was determined using the PSPS containing three sieves (19.00, 8.00, and 1.18 mm) and 1 pan (receive particles <1.18 mm), as previously described (Cao et al., 2013). Physically effective factors (pef) for forage and diets are calculated as the sum of DM proportion retained on the sieves, referring to the ability of a specific kind of feed to stimulate animals to chew, which range from 0 to 1.0 (Zebeli et al., 2012). For example, pef_>1.18_ and pef_>8.00_ equal to the sum of the DM proportion retained on sieves (19.00, 8.00, and 1.18 mm) and the sum of the DM proportion retained on two sieves (19.00 and 8.00 mm). The value of peNDF in the diet is calculated as follows: peNDF (% of diet) =NDF × pef, where NDF presents the percentage of NDF in the diet (Yang and Beauchemin, 2005). Based on this, peNDF_>1.18_ and peNDF_>8.00_ could be calculated as the dietary NDF content multiplied by pef_>1.18_ and pef_>8.00_, respectively.

In this study, we distinguished the five treated diets based on the difference in the content of peNDF_>1.18_, as fed <1.18 mm in length can pass through the rumen to the following digestive tract (Maulfair et al., 2011), while the feed particles retained on the 1.18 mm sieve have high resistance to passing through the rumen, resulting in increased chewing and rumination activities (Poppi et al., 1981). The concentrates and forages were mixed and stirred on a total mixed ration (TMR) machine (Shandong Shengshi Machinery Manufacturing Co., Ltd, Qufu, Shandong) for 2 min before feeding. TMR was supplied to the goats twice daily at 08: 00 and 20: 00 for *ad libitum* intake (at least 10% orts). Clean water was available *ad libitum* throughout the study.

All the animals were fed in a single cage for a 30-day formal feeding trial, after a 14-d adaptation period. Total collection of feces was conducted on day 7 of the formal period for six consecutive days till day 12. On days 13 to 28, three goats in each treatment were selected for methane (CH_4_) measurements in four separate open-circuit respiratory chambers. These goats stayed in chambers for four consecutive days with a 2-day adaptation period and a 2-day sampling and measurement period.

### Sample collection

Before feeding on days 7 to 12, total fecal samples were collected by grab sampling two times daily, weighed, and recorded. A 10% aliquot of each fecal sample was removed and immediately stored in a freezer (−20°C) until further analysis. At the end of the feeding trial (day 30), 15 goats (three goats from each treatment) were slaughtered after electric shock and the others were continually kept in pens. Samples of rumen fluid and rumen chyme were collected from rumen immediately after slaughtering. Approximately 50 ml of rumen fluid was collected from each goat by filtering rumen content through four layers of gauze and then divided into two aliquots after pH determination using a PHS-3B acidometer (Shanghai INESA Scientific Instrument Co., Ltd., Shanghai, China). One aliquot was transferred into sterilized tubes and placed in liquid nitrogen as soon as possible and was taken to the laboratory and stored in a refrigerator at −80°C for DNA extraction. Another aliquot was aliquoted and transferred into sterilized tubes and stored at −80°C for chemical assay of volatile fatty acids (VFAs), ammonia nitrogen (N-NH_3_), and microbial crude protein (MCP) concentrations.

### Methane emission measurements

An open-circuit respiratory chamber was used for CH_4_ measurements, and the CH_4_ production was calculated as described previously (Aguilera and Prieto, 1986). Three goats in each treatment were selected for CH_4_ measurements, with one goat in each chamber. The total volume of 6 m^3^ (2.5 m long, 1.5 m wide, and 1.6 m high) of each chamber was ventilated by suction pumps set a range of 16–20 m^3^/h, allowing a slight negative pressure within the chambers. Temperature and relative humidity were set at 25 ± 1°C and 60 ± 10% respectively with air conditioning units. The CH_4_ concentration in the air entering and leaving each individual chamber was measured every 10 minutes by using an MGA3000 Multi-Gas Analyzer (ADC Gas Analysis Ltd., Hoddesdon, United Kingdom). The analyzer was calibrated weekly using oxygen-free nitrogen (zero gas) and a known quantity of CH_4_ (span gas). The flow measurement systems were checked before and immediately after the experiment by releasing analytical grade CH_4_ into the chambers, by determining the recovery of CH_4_. The purpose of the calibrations was to ensure a recovery rate of CH_4_ at a range of 97 to 103%. The concentration of CH_4_ was analyzed using gas chromatography. Each chamber contained a feed bin, drinking water container, and separate trays.

### Chemical analysis

The contents of DM, organic matter (OM), and crude protein (CP), in feed samples and fecal samples, were analyzed according to AOAC (International, 1990), and the contents of neutral detergent fiber (NDF) and acid detergent fiber (ADF) in these samples were determined according to previously described (Van Soest, 1963). Subsequently, the apparent digestibility of DM, OM, CP, NDF, and ADF were calculated through the formula: apparent nutrient digestibility =100% × [(feed intake × nutrient content in the diet)—(fecal output × nutrient content in the feces)]/ feed intake × nutrient content in the diet (Almeida and Stein, 2010).

The concentration of VFAs in rumen fluid was analyzed with a Varian CP-3800 gas chromatograph (Agilent Technologies, Santa Clara, USA). The thawed rumen fluid samples were centrifuged at 500 × g for 15 min, 0.2 ml of the 25% metaphosphoric acid solution (**w/v**) and 23.3 μl of the 210 mmol/L crotonic acid solution were added to 1 mL of the supernatant. The mixed solution was incubated at 4°C for 30 min and then centrifuged at 16,000 × g for 10 min to obtain new supernatants. Subsequently, each supernatant sample (0.3 ml) was diluted 4-fold (v/v) with chromatographic grade methanol. In subsequent experiments, 1 μl of the rumen fluid sample was injected using a 50:1 split ratio at 220°C. The chromatographic column was programmed from 100 to 190°C at 20°C/min. Nitrogen was employed as the carrier gas at a constant flow of 1 ml/min. The concentration of NH_3_-N was measured using UV-3600i Plus spectrophotometrically (Shimadzu, Kyoto, Japan) according to the method described previously (Weatherburn, 1967). Briefly, the thawed samples of the rumen fluid were centrifuged (6,500 × g) at 4°C for 15 min, 2.5 ml of the prepared chromogenic solution (10 g of phenol and 50 mg of sodium nitroprusside to 1 L of solution) and 2 ml of the alkaline hypochlorite (5 g of sodium hydroxide with 50 ml of 5.25% sodium hypochlorite (w/v) per liter of solution) were added to 80 μl of the supernatant. The absorbance was read at the wavelength of 625 nm after the mixed solution was incubated at 37°C for 20 min to calculate the concentration of NH_3_-N. The microbial protein (MCP) in rumen fluid was obtained by repeated centrifugation as previously reported (Berthiaume et al., 2010) and the concentration of MCP was determined by using bicinchoninic acid (BCA) protein concentration kit (Nanjing Jiancheng Bioengineering Institute, Nanjing, China). In brief, the thawed samples of the rumen fluid were centrifuged at 500 × g and 4°C for 10 min, then the supernatant was centrifuged at 20,000 × g and 4°C for 30 min. The precipitate was washed with deionized water and centrifuged at 20,000 × g and 4°C for 30 min, repeated 3 times. The final pellet was thoroughly diluted with deionized water, and 10 μl of the dilution was blended with 250 μl reaction fluid at 37°C for 30 min. The MCP content could be calculated by measuring the optical density value at 562 nm in a SpectraMax M5 microplate reader (Molecular Devices, Sunnyvale, CA, USA) according to the manufacturer's instructions.

### DNA extraction and sequencing

Total DNA was extracted from each sample by using the FastPure^®^ Microbiome DNA Isolation Kit (Tiangen Biotech Co., Ltd., Beijing, China) according to the protocol provided by the manufacturer. The DNA was quantified by using the NanoDrop^®^ ND-2000 micro-spectrophotometer (Thermo Scientific, Wilmington, DE, USA). The integrity of DNA samples was assessed with 0.8% denatured agarose gel electrophoresis. The specific primers with barcode were used to amplify the V4-V5 region of the 16S ribosomal RNA (rRNA) gene of archaea, and the primer sequences were as follows: 519F (5′-CAGCMGCCGCGGTAA-3′) and 976R (5′-CCGGCGTTGAMTCCAATT-3′) (Teske and Sørensen, 2008). Similarly, the sequences of the primers used to amplify the fungal internal transcribed spacer (ITS) 2-rDNA region were as follows: ITS3_KYO2 (5′-GATGAAGAACGYAGYRAA-3′) and ITS4 (5′-TCCTCCGCTTATTGATATGC-3′) (Toju et al., 2012). The amplification was performed by GeneAmp^®^ PCR System 9700 (Applied Biosystems, Inc., Foster City, CA, USA). The amplification conditions of archaea were 94°C for 1 min; 94°C for 20 s, 54°C for 30 s, 72°C for 30 s, repeated for 30 cycles; 72°C for 5 min. The amplification conditions of fungi were 94°C for 1 min; 94°C for 20 s, 48°C for 30 s, 72°C for 30 s, repeated for 30 cycles; 72°C for 5 min. The PCR product of each sample was detected by 2% agarose gel electrophoresis and sent to Rhonin Biotechnology Co., Ltd (Chengdu, China) for high-throughput sequencing analysis on the Illumina HiSeq 2500 (PE250) platform after fluorescent quantization.

The original offline data obtained by sequencing was spliced and filtered to obtain a high-quality target sequence for subsequent analysis. Subsequent bioinformatics operations were completed using Usearch and QIIME4 (Edgar, 2013). Statistics and mapping are mainly done using R5, python, and java. The main steps were as follows: stitching the sequences, distinguishing samples according to barcode, removing the low quality sequences, removing the chimera, clustering the operational taxonomic units (OTUs) at a similarity of 97%, selecting the representative sequence of OTUs, classifying the species using the SILVA ribosomal RNA database (http://www.arb-silva.de/) (Quast et al., 2012) and the UNITE database (https://unite.ut.ee/) (Kõljalg et al., 2013), aligning and filtering the representative sequences and then reconstructing the phylogenetic tree, filtering out the unwanted OTUs and resampling, calculating the abundance at each classification level, analyzing the community composition, analyzing the alpha diversity and beta diversity, differentiating the species, and analyzing the correlation between species and functions. For the functional classification prediction of fungal communities, we used FUNGuild (an open annotation tool for parsing fungal community datasets by ecological guild, https://github.com/UMNFuN/FUNGuild) to parse fungal OTUs into trophic modes and guilds (Toju et al., 2016).

### Statistical analysis and calculation

The experimental data in this study were statistically analyzed using one-way analysis of variance (ANOVA) followed by Tukey's post hoc test or Kruskal–Wallis-test with SPSS version 26.0 (SPSS Inc., Chicago, IL, USA). The quadratic regression analysis was used to analyze the relationship between the nutrient digestibility and the level of dietary peNDF.Spearman correlation coefficients (r) and *p* value were analyzed using the OmicShare tools (a free online platform for data analysis, https://www.omicshare.com/tools) to show correlations between the feed intake, digestibility, rumen fermentation, methane production with the relative abundance of fungi genera and methanogenic species. *p* < 0.05 was accepted as statistically significant differences. Results are presented as mean and standard error.

## Results

### Levels of dietary physically effective neutral detergent fiber

As shown in Table 2, the values of pef_>8.00_ in five treatments could be calculated as 36.97, 22.95, 16.25, 6.27, and 3.12% according to the proportions of the particles retained on 19.00 mm and 8.00 mm sieves; the values of peNDF _>8.00_ in five treatments could be calculated as 15.86, 9.84, 6.97, 2.69, and 1.34% according to the values of pef_>8.00_ and the percentage of NDF in the diet (42.89%, Table 1). Similarly, the values of pef_>1.18_ in five treatments could be calculated as 76.88%, 69.78%, 65.61%, 61.73%, and 57.71% according to the proportions of the particles retained on 19.00, 8.00, and 1.18 mm sieves; the values of peNDF_>1.18_ in five treatments could be calculated as 32.97%, 29.93%, 28.14%, 26.48%, and 24.75%, respectively.

**Table 2:** Particle size distribution, physical effectiveness factors, and physically effective neutral detergent fiber content of the treatment rations fed to goats (DM basis).

**Items**	**Dietary peNDF**_>1.18^a^_				
	**32.97%**	**29.93%**	**28.14%**	**26.48%**	**24.75%**
Particle separator of TMR^b^					
Particles > 19.00 mm (%)	27.25	11.17	6.98	0	0
Particles 8.00 to 19.00 mm (%)	9.72	11.78	9.27	6.27	3.12
Particles 1.18 to 8.00 mm (%)	39.91	46.83	49.36	55.46	54.58
Particles <1.18 mm	23.12	30.22	34.39	38.27	42.3
Physical effectiveness factor^c^					
pef_>8.00_ (%)	36.97	22.95	16.25	6.27	3.12
pef_>1.18_ (%)	76.88	69.78	65.61	61.73	57.71
peNDF content of DM^d^					
peNDF_>8.00_ (%)	15.86	9.84	6.97	2.69	1.34
peNDF_>1.18_ (%)	32.97	29.93	28.14	26.48	24.75

a Different dietary peNDF_>1.18_ (particle size >1.18 mm) contents of 32.97, 29.93, 28.14, 26.48, and 24.75% were obtained by chopping or crusher crushing the forage into the following lengths: long (7 cm), medium (4 cm), short (1 cm), fine (5 mm sieve), very fine (1 mm sieve).

b Particle length of ration variables was measured using the Penn State Particle Separator.

c pef_>8.0_ and pef_>1.18_, physical effectiveness factor determined as the proportion of whole samples particles retained on 2 sieves or on 3 sieves, respectively.

d peNDF_>8.00_ and peNDF_>1.18_, physically effective NDF determined as NDF content of ration sample multiplied by pef_>8.00_ and pef_>1.18_, respectively.

### Apparent digestibility of nutrients

Apparent digestibility of DM, OM, CP, NDF, and ADF varied greatly (*p* < 0.05) among the treatment groups (Table 3), the highest and the lowest digestibility of those nutrients were observed in the 28.14% and 24.75% peNDF_>1.18_ treatments, respectively. Regression analysis showed that the digestibility of DM, OM, NDF, and ADF was quadratically correlated with dietary peNDF_>1.18_ levels (Table 4). It could be calculated that the highest digestibility of DM, OM, NDF, and ADF appeared at dietary peNDF_>1.18_ levels of 30.25%, 30.18%, 30.36%, and 30.67%.

**Table 3:** Effects of different contents of peNDF_>1.18_ in diets on apparent nutrient digestibility in goats.

**Item**	**Dietary peNDF**_>1.18^1^_					**SEM**	***p*** **-value**
	**32.97%**	**29.93%**	**28.14%**	**26.48%**	**24.75%**		
Intake (g)							
peNDF_>8.00_	107.53^a^	69.27^b^	51.37^c^	22.35^d^	9.41^e^	5.14	< 0.001
peNDF_>1.18_	223.54^a^	210.71^b^	207.39^b^	220.05^ab^	173.75^c^	8.93	< 0.001
Apparent nutrient digestibility (%)							
Dry matter	66.07^a^	64.38^ab^	68.03^a^	65.00^a^	53.07^b^	1.71	0.019
Organic matter	72.80^a^	71.11^ab^	78.90^a^	68.04^ab^	59.00^b^	2.03	0.006
Crude protein	64.14^b^	63.25^b^	73.06^a^	63.56^b^	62.29^b^	1.89	0.016
Neutral detergent fiber	63.53^ab^	67.83^a^	67.82^a^	53.33^b^	39.92^c^	3.84	< 0.001
Acid detergent fiber	65.90^a^	63.29^ab^	70.10^a^	53.89^b^	42.37^c^	3.61	< 0.001

1 Different dietary peNDF_>1.18_ (particle size >1.18 mm) contents of 32.97, 29.93, 28.14, 26.48, and 24.75% were obtained by chopping or crusher crushing the forage into the following lengths: long (7 cm), medium (4 cm), short (1 cm), fine (5 mm sieve), very fine (1 mm sieve).

Different letters^a−c^ denote p < 0.05.

**Table 4:** Regression equations between dietary peNDF_>1.18_ levels (x) and (y) (*n* =30, R^2^> 0.55).

**Items**	**Regression model**	**R^2^**	***p*** **-value**
DM (%)	y = −0.441x^2^ + 26.679x – 334.996	0.654	0.002
OM (%)	y = −0.557x^2^ + 33.619x – 430.9	0.558	0.007
NDF (%)	y = −0.954x^2^ + 57.927x – 810.075	0.733	<0.001
ADF (%)	y = −0.727x^2^ + 44.588x – 615.365	0.602	0.004

### Ruminal fermentation parameters and methane production

The effects of dietary peNDF_>1.18_ content on rumen fermentation parameters and methane production are presented in Table 5. Dietary peNDF_>1.18_ content did not affect ruminal pH and the concentration of VFAs. Ruminal NH_3_-N concentrations in the 29.93% and 28.14% groups were higher than that in the 24.75% group (*p* < 0.05). Ruminal MCP concentration was the highest in the 32.97% group (*p* < 0.05). Daily CH_4_ production in the 32.97% and 24.75% peNDF_>1.18_ treatments was lower than that in the 26.48% group (*p* < 0.05) and no differences were observed among other groups. CH_4_ production did not differ among the groups when expressed as per unit of DM intake.

**Table 5:** Effects of different contents of peNDF_>1.18_ in diets on ruminal fermentation characteristics in goats.

**Items**	**Dietary peNDF**_>1.18^1^_					**SEM**	***p*** **-value**
	**32.97%**	**29.93%**	**28.14%**	**26.48%**	**24.75%**		
CH_4_ (L/d)	15.03^b^	16.12^ab^	16.37^ab^	17.93^a^	14.68^b^	0.32	0.003
CH_4_/DMI (L/kg)	22.84	23.53	22.52	22.38	21.52	0.25	0.431
Ruminal pH	6.1	6.2	6.2	6.3	6.1	0.07	0.263
Total VFAs (mmol/L)	76.30	78.57	83.42	85.28	83.10	4.29	0.476
Acetate (mmol/L)	54.61	56.05	60.39	61.98	59.03	2.94	0.376
Propionate (mmol/L)	12.38	13.57	14.63	14.59	14.05	0.33	0.295
Butyrate (mmol/L)	9.32	8.96	8.40	8.71	10.01	0.37	0.846
Acetate/Propionate	4.43	4.16	4.16	4.25	4.21	0.23	0.909
NH_3_-N (mg/100 mL)	16.40^ab^	19.45^a^	19.70^a^	14.17^ab^	11.18^b^	1.06	0.020
MCP (mg/mL)	2.57^a^	2.40^b^	2.34^b^	2.28^b^	2.26^b^	0.04	0.009

1 Different dietary peNDF_>1.18_ (particle size >1.18 mm) contents of 32.97, 29.93, 28.14, 26.48, and 24.75% were obtained by chopping or crusher crushing the forage into the following lengths: long (7 cm), medium (4 cm), short (1 cm), fine (5 mm sieve), very fine (1 mm sieve).

Different letters^a, b^ denote p < 0.05.

### Sequencing data and abundance of archaea

**Figure 1:**
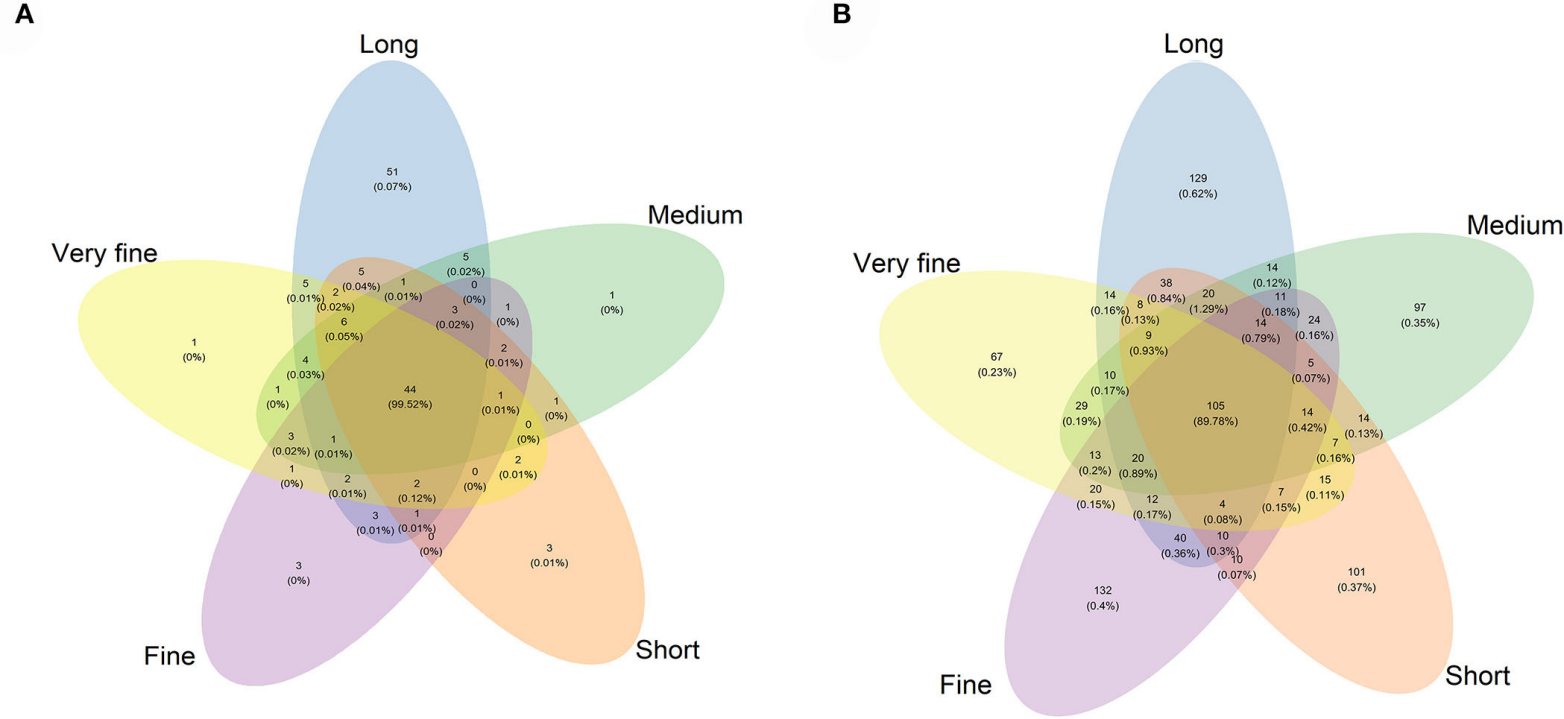
Venn diagram of the OTUs among the five treatments of archaea **(A)** and fungi **(B)**. Long: 32.97% peNDF_>1.18_ treatment; Medium: 29.93% peNDF_>1.18_ treatment; Short: 28.14% peNDF_>1.18_ treatment; Fine: 26.48% peNDF_>1.18_ treatment; Very fine: 24.75% peNDF_>1.18_ treatment.

A total of 482,535 effective sequences that met quality control were obtained from the 16S rRNA high-throughput sequencing analysis. Based on 97% sequence similarity from valid sequences, these sequences were completely clustered into 876 OTUs with a mean of 58.4 ± 17.8 (mean ± SD, *n* = 3) OTUs per sample. The average number of OTUs, Chao1 index, Shannon index, and Simpson index were the highest in the 32.97% peNDF_>1.18_ treatment (*p* < 0.05), and the Shannon index and Simpson index were the lowest in the 29.93, 28.14, and 26.48% peNDF_>1.18_ treatments (Table 6). A total of 44 OTUs were shared across the five treatments, and the number of sequences in shared OTUs accounted for 99.52% of the total effective sequences (Figure 1A).

**Table 6:** Effects of different contents of peNDF_>1.18_ in diets on rumen microbial OTUs and alpha diversity of goats.

**Items**	**Dietary peNDF**_>1.18^1^_					**SEM**	***p*** **-value**
	**32.97%**	**29.93%**	**28.14%**	**26.48%**	**24.75%**		
16S rRNA gene sequence analysis							
Effective sequences	30,923	31,948	30,293	30,977	33,370	743.48	0.779
OUT number	87.00^a^	52.67^b^	51.00^b^	46.67^b^	54.67^b^	4.59	0.008
Chao1	126.89^a^	64.20^ab^	57.98^ab^	53.95^b^	65.50^ab^	9.24	0.038
Shannon	1.30^a^	0.61^c^	0.56^c^	0.60^c^	0.82^b^	0.07	< 0.001
Simpson	0.62^a^	0.23^c^	0.20^c^	0.23^c^	0.31^b^	0.04	< 0.001
ITS sequence analysis							
Effective sequences	32,721	33,025	33,115	33,258	34,944	827.65	0.948
OUT number	458^a^	406^bc^	381^c^	441^b^	354^c^	80.59	< 0.001
Chao1	422.6^ab^	404.0^b^	357.2^bc^	482.3^a^	333.0^c^	19.96	< 0.001
Shannon	3.910^a^	3.051^b^	3.165^b^	3.113^b^	2.644^c^	0.077	< 0.001
Simpson	0.939^a^	0.837^b^	0.854^b^	0.835^b^	0.728^c^	0.008	< 0.001

1 Different dietary peNDF_>1.18_ (particle size >1.18 mm) contents of 32.97, 29.93, 28.14, 26.48, and 24.75% were obtained by chopping or crusher crushing the forage into the following lengths: long (7 cm), medium (4 cm), short (1 cm), fine (5 mm sieve), very fine (1 mm sieve).

Different letters^a−c^ denote p < 0.05.

**Figure 2:**
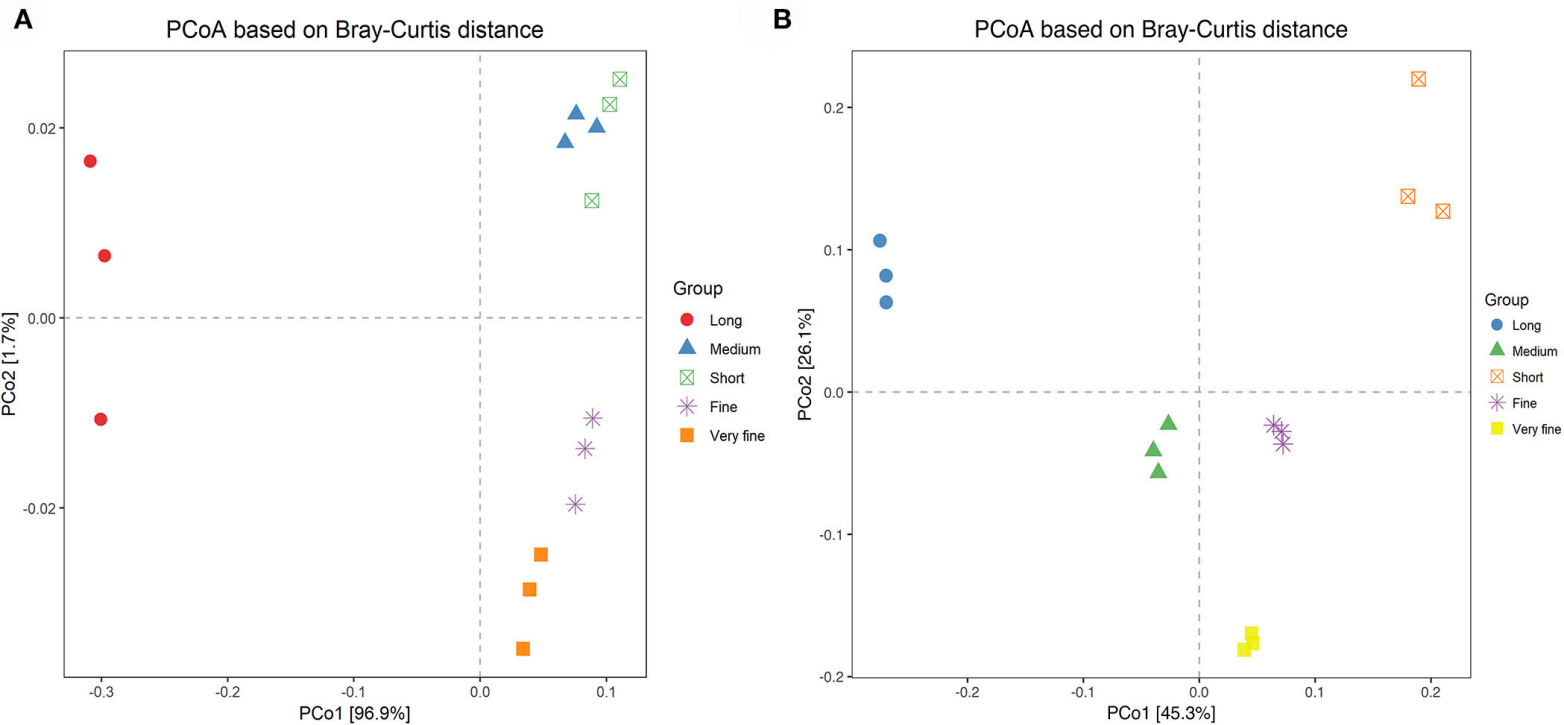
PCoA analysis based on Brary-Curtis distance among the five treatments of archaea **(A)** and fungi **(B)**. Distances between the samples, based on similarity in OTU composition (OTU similarity ≥97%) calculated with Brary-Curtis distance, were visualized by principal coordinates analysis (PCoA) plots. A greater distance between two samples inferred a lower similarity. The percentage of variation explained by PC1 and PC2 were indicated in the axis.

The principal co-ordinate analysis (PCoA) was conducted based on Bray-Curtis distance as shown in Figure 2A. Each treatment was evenly distributed among the clusters. All samples tended to cluster together in accordance with their own ratio treatment. These results suggested strong differences in the structure of archaea among the five treatments. Moreover, the samples of 26.48% and 24.75% peNDF_>1.18_ treatments were clustered in the same area, indicating that the archaeal community structure of the two treatments was more similar to that of other treatments. Similar results appeared in the 29.93% and 28.14% peNDF_>1.18_ treatments.

**Figure 3:**
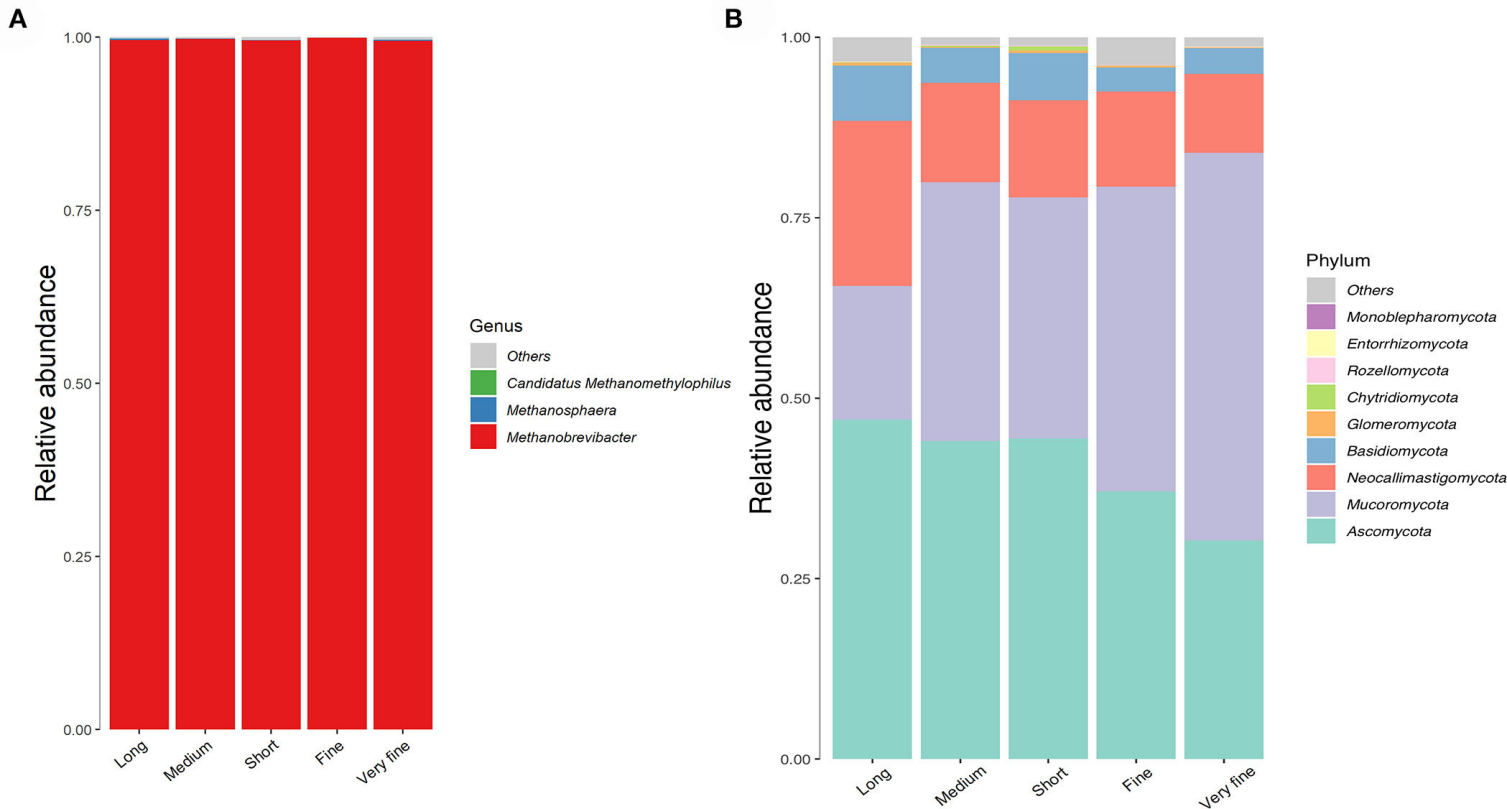
The histogram of the relative abundance of archaea at genus level **(A)** and fungi at phylum level **(B)**. Long: 32.97% peNDF_>1.18_ treatment; Medium: 29.93% peNDF_>1.18_ treatment; Short: 28.14% peNDF_>1.18_ treatment; Fine: 26.48% peNDF_>1.18_ treatment; Very fine: 24.75% peNDF_>1.18_ treatment.

Only the phylum Euryarchaeota of archaea was identified in all samples in this study. A total of four genera of archaea were identified in all samples, with the largest proportion of *Methanobrevibacter* accounting for 99.64%, followed by *F_Methanomethylophilaceae|g_ Uncultured* at 0.25%, *Methanosphaera* at 0.11% and *Candidatus methanomethylophilus* at 0.0034% (Figure 3A, Table 7). The relative abundance of *Methanobrevibacter* was higher in the 26.48% peNDF_>1.18_ treatment than that in the 24.75% peNDF_>1.18_ treatment (*p* < 0.05). The relative abundance of *Methanosphaera* was higher in the 32.97% and 24.75% peNDF_>1.18_ treatments than that in the 29.93%, 28.14%, and 26.48% peNDF_>1.18_ treatments (*p* < 0.05).

**Table 7:** Effects of different contents of peNDF_>1.18_ in diets on rumen archaea abundance in goats.

**Items**	**Dietary peNDF**_>1.18^1^_					**SEM**	***p*** **-value**
	**32.97%**	**29.93%**	**28.14%**	**26.48%**	**24.75%**		
Genus (%)							
*Methanobrevibacter*	99.60^ab^	99.75^ab^	99.50^ab^	99.88^a^	99.46^b^	0.05	0.042
*Methanosphaera*	0.20^a^	0.05^bc^	0.07^bc^	0.04^c^	0.18^ab^	0.02	0.007
Species (%)							
*Methanobrevibacter sp. YE315*	52.91^c^	88.62^a^	90.42^a^	88.06^a^	83.91^b^	3.78	<0.001
*Methanobrevibacter boviskoreani JH1*	35.74^a^	5.29^b^	3.95^c^	2.42^d^	4.68b^c^	3.40	<0.001
*Haemonchus placei*	8.39^a^	4.16^b^	3.63^b^	7.58^a^	8.17^a^	0.57	<0.001
*Methanobrevibacter sp. G16*	1.16^ab^	0.99^b^	0.77^bc^	0.36^c^	1.61^a^	0.12	<0.001
*Methanobrevibacter millerae*	1.30^a^	0.58^b^	0.54^b^	0.93^ab^	0.79^b^	0.08	0.001
Uncultured (%)							
*f__Methanomethylophilaceae|g_uncultured*	0.20	0.20	0.42	0.08	0.37	0.05	0.114

1 Different dietary peNDF_>1.18_ (particle size >1.18 mm) contents of 32.97, 29.93, 28.14, 26.48, and 24.75% were obtained by chopping or crusher crushing the forage into the following lengths: long (7 cm), medium (4 cm), short (1 cm), fine (5 mm sieve), very fine (1 mm sieve).

Different letters^a−d^ denote p < 0.05.

**Figure 4:**
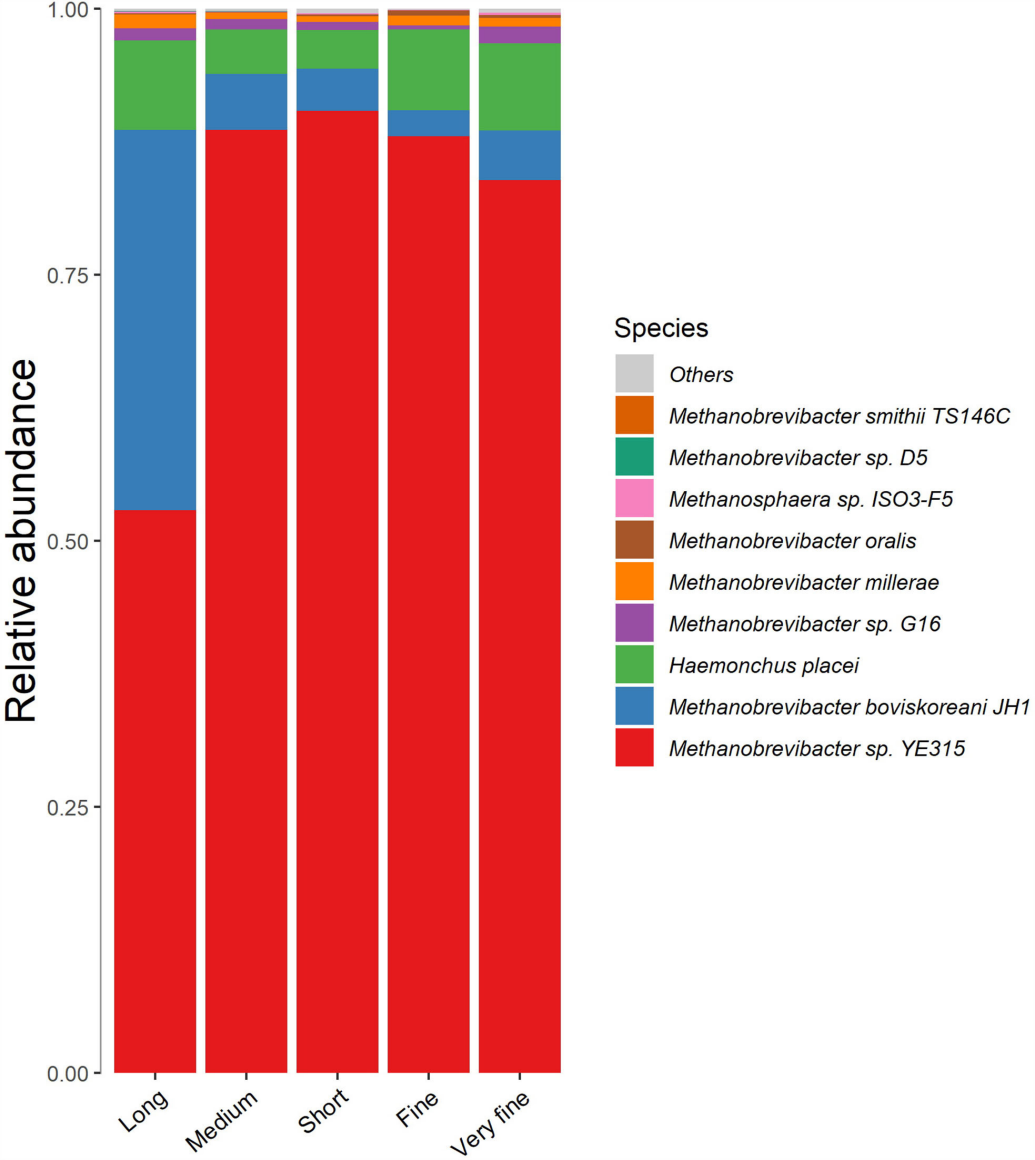
The cluster histogram of methanogen at species level. Long: 32.97% peNDF_>1.18_ treatment; Medium: 29.93% peNDF_>1.18_ treatment; Short: 28.14% peNDF_>1.18_ treatment; Fine: 26.48% peNDF_>1.18_ treatment; Very fine: 24.75% peNDF_>1.18_ treatment.

The archaea varied greatly with treatments at the species level (Figure 4). The dominant species for methanogenesis was *Methanobrevibacter sp. YE315*, followed by *Methanobrevibacter boviskoreani JH1*. The abundance and diversity of archaea are similar between the groups of consecutive peNDF levels, while the 32.97% peNDF_>1.18_ treatment was quite different from other treatments. The relative abundance of *Methanobrevibacter sp. YE315* was lower in the 32.97% peNDF_>1.18_ treatment than that in other treatments (*p* < 0.05). The relative abundance of *M. boviskoreani JH1* was higher in the 32.97% peNDF_>1.18_ treatment than that in other treatments (*p* < 0.05).

### Sequencing data and abundance of fungi

A total of 501,190 valid sequences that met quality control were obtained from the fungal ITS sequencing analysis. Based on 97% sequence similarity from valid sequences, these sequences were completely clustered into 1,013 OTUs with a mean of 301 ± 33.7 (mean ± SD, n = 3) OTUs per sample. The average number of ruminal fungal OTUs was the greatest in the 32.97% peNDF_>1.18_ treatment (*p* < 0.05), and the lowest value was observed in the 28.14% peNDF_>1.18_ and 24.75% peNDF_>1.18_ treatments (Table 6). Both the Shannon-Wiener index and Simpson index in the 32.97% peNDF_>1.18_ treatment were higher than that in other treatments, and in the 24.75 % peNDF_>1.18_ treatment was lower than that in other treatments (*p* < 0.05). A total of 105 OTUs were shared across the five treatments, and the number of sequences in shared OTUs accounted for 89.78% of the total effective sequences (Figure 1B).

The principal co-ordinate analysis (PCoA) was conducted based on the Euclidean distances between the different treatments, and the results are shown in Figure 2B. Each treatment was evenly distributed among the clusters. All samples tended to cluster together in accordance with their own ration treatment. These results suggested strong differences in the fungal structure among the treatments. Moreover, the samples of 24.75% and 26.48% peNDF_>1.18_ treatment were clustered in the same area, indicating that the fungal community structure of the two treatments was more similar to that of other treatments.

A total of 10 fungal phyla were identified in all samples, seven of which were shared (Figure 3B). The top four most abundant phyla ranked in descending order were Ascomycota, Mucoromycota, Neocallimastigomycota, and Basidiomycota, accounting for an average of 40.6, 36.8, 14.9, and 5.2% of the total fungi at the phyla level, respectively. In addition to Basidiomycota, the fungal community at the phylum level differed significantly among the treatments (*p* < 0.05). The relative abundance of Ascomycota was lowest in the 24.75% peNDF_>1.18_ treatment, followed by the 26.48% peNDF_>1.18_ treatment (*p* < 0.05), while the relative abundance of Mucoromycota was lowest in the 24.75% peNDF_>1.18_ treatment, followed by the 26.48% peNDF_>1.18_ treatment (*p* < 0.05). The relative abundance of Neocallimastigomycota and Glomeromycota were higher in the 32.97% peNDF_>1.18_ treatment than that in other treatments (*p* < 0.05).

At the genus level, a total of 186 fungal genera were detected, 14 of which were detected in all goats with an average abundance of >1%, including *o_GS23|f_uncultured, f_Saccharomycetales_fam_Incertae_sedis|g_uncultured, Candida, f_Neocallimastigaceae|g_uncultured, Neocallimastix, Symbiotaphrina, Orpinomyces, o_Pleosporales|f_uncultured, o_Agaricostilbales|f_uncultured, o_Sebacinales|f_uncultured, f_Phaeosphaeriaceae|g_uncultured, Yarrowia, Aspergillus, p_Ascomycota|c_uncultured* (Table 8, Supplementary Figure S1). The top 50 most abundant fungal genera were displayed in heatmap (Supplementary Figure S2). The dominant fungi at the genus level in the rumen of goats were *Candida, Neocallimastix*, and *Orpinomyces*, accounting for an average of 8.92% and 5.49% of the total fungi at the genus level, respectively. All the fungi at the genus level differed greatly among the treatments (*p* < 0.05). The relative abundance of *Candida* went up at first and then dropped with the decrease of dietary peNDF_>1.18_ content from 32.97 to 24.75%, and reached the maximum at the content of peNDF_>1.18_ was 28.1%. The relative abundance of *Neocallimastigomycota Neocallimastix* was the highest in the 29.93% peNDF_>1.18_ treatment, followed by the 32.97% peNDF_>1.18_ treatment.

**Table 8:** Effects of different contents of peNDF_>1.18_ in diets on rumen fungi abundance in goats.

**Items**	**Dietary peNDF**_>1.18^1^_					**SEM**	***P*** **-value**
	**32.97%**	**29.93%**	**28.14%**	**26.48%**	**24.75%**		
Phylum (%)							
Ascomycota	47.05^a^	44.02^a^	44.38^a^	37.09^b^	30.27^c^	1.413	< 0.001
Mucoromycota	18.50^d^	35.90^c^	33.45^c^	42.20^b^	53.75^a^	1.302	< 0.001
Neocallimastigomycota	22.91^a^	13.80^b^	13.46^b^	13.25^b^	10.93^b^	1.359	< 0.001
Basidiomycota	7.63	4.84	6.55	3.30	3.55	0.685	0.188
Glomeromycota	0.41^a^	0.15^c^	0.34^ab^	0.24^bc^	0.11^c^	0.032	< 0.001
Genus (%)							
*Candida*	5.068^c^	4.560^c^	21.419^a^	11.220^b^	2.308^d^	0.531	< 0.001
*Yarrowia*	2.145^a^	1.185^b^	0.914^b^	1.061^b^	1.275^b^	0.157	< 0.001
*Rhexocercosporidium*	0.852^a^	0.705^ab^	0.593^bc^	0.587^bc^	0.468^c^	0.055	< 0.001
*Cercospora*	0.801^a^	0.514^b^	0.293^b^	0.536^ab^	0.497^b^	0.083	0.002
*Aureobasidium*	0.689^a^	0.412^ab^	0.248^b^	0.564^a^	0.474^ab^	0.087	0.005
*Macrorhabdus*	0.322^ab^	0.288^b^	0.502^a^	0.237^b^	0.248^b^	0.059	0.007
*Fusarium*	0.442^a^	0.248^b^	0.316^ab^	0.333^ab^	0.231^b^	0.050	0.013
*Batcheloromyces*	0.339^a^	0.220^ab^	0.226^ab^	0.135^b^	0.237^ab^	0.044	0.014
*Neocallimastix*	6.660^ab^	7.399^a^	5.971^b^	4.035^c^	3.398^c^	0.379	0.013
Uncultured (%)							
*o_GS23|f_uncultured*	17.429^d^	35.625^c^	33.339^c^	42.019^b^	53.386^a^	1.301	< 0.001
*f_Saccharomycetales_fam_Incertae_sedis|g_uncultured*	16.125^b^	22.282^a^	7.269^d^	9.504^d^	12.625^c^	0.773	0.009
*o_Pleosporales|f_uncultured*	3.550^a^	2.173^bc^	1.484^d^	2.551^b^	1.682^cd^	0.182	0.010
*f_Didymellaceae|g_uncultured*	0.988^a^	0.813^ab^	0.677^b^	0.649^b^	0.672^b^	0.084	0.011
*f_Neocallimastigaceae|g_uncultured*	11.243^a^	3.488^bc^	2.314^c^	5.401^b^	4.024^bc^	0.660	< 0.001
*o_Sebacinales|f_uncultured*	2.946^a^	2.196^b^	1.501^c^	2.331^b^	2.088^b^	0.137	< 0.001

1 Different dietary peNDF_>1.18_ (particle size >1.18 mm) contents of 32.97, 29.93, 28.14, 26.48, and 24.75% were obtained by chopping or crusher crushing the forage into the following lengths: long (7 cm), medium (4 cm), short (1 cm), fine (5 mm sieve), very fine (1 mm sieve).

Different letters^a−d^ denote p < 0.05.

### Functional prediction of rumen archaea and fungi

The functional information of rumen archaea in each treatment based on the functional prediction of archaea by the Tax4Fun program and the SILVA database is summarized in Table 9. We found that the functional prediction of rumen archaea was significantly affected by dietary peNDF_>1.18_ levels. The main gene functions of rumen archaea were associated with amino acid metabolism, carbohydrate metabolism, membrane transport, and replication and repair. The functional classification prediction of rumen fungi in each treatment based on the OTU abundance is shown in Table 10. The rumen fungal functional guilds were significantly affected by dietary peNDF_>1.18_ levels. The trophic mode revealed that the most diverse fungal type was the pathotrophs. Meanwhile, the guild classification implied that the most diverse guilds were animal pathogens.

**Table 9:** Effects of dietary peNDF_>1.18_ level on the functional prediction of rumen archaea (%).

**Items**	**Dietary peNDF**_>1.18^1^_					**SEM**	***p*** **-value**
	**32.97%**	**29.93%**	**28.14%**	**26.48%**	**24.75%**		
Metabolism							
Amino acid metabolism	10.258^a^	10.186^ab^	10.178^b^	10.184^ab^	10.048^c^	0.019	< 0.001
Biosynthesis of other secondary metabolites	1.066^b^	1.074^b^	1.097^a^	1.069^b^	1.003^c^	0.009	< 0.001
Carbohydrate metabolism	10.109^d^	10.234^ab^	10.285^a^	10.183^bc^	10.163^c^	0.017	< 0.001
Energy metabolism	6.009^a^	6.002^a^	6.034^a^	6.059^a^	5.828^b^	0.023	< 0.001
Enzyme families	2.231^a^	2.236^a^	2.233^a^	2.229^a^	2.199^b^	0.004	< 0.001
Glycan biosynthesis and metabolism	2.704^a^	2.712^a^	2.716^a^	2.735^a^	2.415^b^	0.033	< 0.001
Lipid metabolism	2.745^b^	2.702^c^	2.740^b^	2.738^b^	2.829^a^	0.012	< 0.001
Metabolism of cofactors and vitamins	4.619^a^	4.604^a^	4.543^b^	4.583^ab^	4.418^c^	0.020	< 0.001
Metabolism of other amino acids	1.536^b^	1.545^ab^	1.563^a^	1.543^ab^	1.463^c^	0.009	< 0.001
Metabolism of terpenoids and polyketides	1.746^a^	1.755^a^	1.723^bc^	1.738^ab^	1.702^c^	0.005	< 0.001
Nucleotide metabolism	4.344^a^	4.338^a^	4.282^b^	4.343^a^	4.239^c^	0.012	< 0.001
Xenobiotics biodegradation and metabolism	1.593^b^	1.573^b^	1.515^d^	1.546^c^	1.652^a^	0.013	< 0.001
Cellular processes							
Cell growth and death	0.562^b^	0.574^a^	0.558^b^	0.565^b^	0.542^c^	0.003	< 0.001
Cell motility	2.554^b^	2.485^b^	2.728^a^	2.541^b^	2.860^a^	0.039	< 0.001
Transport and catabolism	0.365^a^	0.344^b^	0.370^a^	0.369^a^	0.334^b^	0.004	< 0.001
Environmental information processing					
Membrane transport	9.374^b^	9.550^b^	9.511^b^	9.374^b^	10.242^a^	0.091	< 0.001
Signal transduction	1.489^c^	1.456^c^	1.538^b^	1.491^c^	1.586^a^	0.013	< 0.001
Signaling molecules and interaction	0.172^b^	0.171^b^	0.168^b^	0.171^b^	0.161^a^	0.001	< 0.001
Genetic information processing							
Folding, sorting and degradation	2.588^b^	2.582^b^	2.589^b^	2.609^b^	2.523^a^	0.008	< 0.001
Replication and repair	9.607^a^	9.588^a^	9.502^b^	9.646^a^	9.455^b^	0.020	< 0.001
Transcription	2.699^b^	2.662^bc^	2.631^c^	2.644^bc^	2.830^a^	0.020	< 0.001
Translation	6.144^b^	6.142^b^	6.100^bc^	6.209^a^	6.085^c^	0.012	<0.001
Human diseases							
Cancers	0.106	0.107	0.107	0.106	0.106	0.001	0.179
Cardiovascular diseases	0.213	0.215	0.214	0.213	0.212	0.001	0.159
Infectious diseases	0.245^a^	0.242^ab^	0.240^bc^	0.240^bc^	0.238^c^	0.001	0.001
Metabolic diseases	0.116^b^	0.114^c^	0.117^ab^	0.119^a^	0.115^bc^	0.001	< 0.001
Neurodegenerative diseases	0.111^a^	0.105^b^	0.110^ab^	0.110^ab^	0.107^ab^	0.001	0.028
Organismal systems							
Environmental adaptation	0.151^c^	0.151^c^	0.160^a^	0.154^b^	0.160^a^	0.001	< 0.001
Nervous system	0.107^a^	0.104^bc^	0.103^c^	0.105^b^	0.104^b^	0.001	< 0.001
Unclassified							

1 Different dietary peNDF_>1.18_ (particle size >1.18 mm) contents of 32.97, 29.93, 28.14, 26.48, and 24.75% were obtained by chopping or crusher crushing the forage into the following lengths: long (7 cm), medium (4 cm), short (1 cm), fine (5 mm sieve), very fine (1 mm sieve).

Different letters^a−d^ denote p < 0.05.

**Table 10:** Effects of dietary peNDF_>1.18_ level on the prediction of rumen fungal functional guilds.

**Items**		**Dietary peNDF**_>1.18^1^_					**SEM**	***p*** **-value**
		**32.97%**	**29.93%**	**28.14%**	**26.48%**	**24.75%**		
Trophic mode	Fun guild	Guild OTU richness (%)						
Symbiotroph	Fungal parasite	12.67^b^	11.31^b^	8.25^c^	15.75^a^	10.94^b^	0.68	< 0.001
	Arbuscular mycorrhizal	1.73^a^	0.69^c^	1.21^b^	1.54^a^	0.56^c^	0.12	< 0.001
	Ectomycorrhizal	3.16^a^	0.74^c^	2.01^b^	1.17^a^	2.11^c^	0.23	< 0.001
	Animal endosymbiont	11.95^b^	21.32^a^	10.01^b^	10.89^b^	7.36^c^	1.29	< 0.001
Saprotroph	Wood saprotroph	14.73^b^	12.76^b^	14.30^b^	20.64^a^	9.99^b^	1.01	< 0.001
	Soil saprotroph	6.17^b^	6.08^b^	5.75^b^	9.33^a^	4.92^b^	0.46	0.002
	Undefined saprotroph	3.04^a^	2.35^b^	1.49^c^	2.86^a^	2.15^b^	0.15	< 0.001
Pathotroph	Animal pathogen	47.40^ab^	38.34^bc^	28.62^c^	53.06^a^	37.36^bc^	2.42	< 0.001
	Plant pathogen	4.42^ab^	4.34^b^	3.48^b^	5.95^a^	3.58^b^	0.27	< 0.001

1 Different dietary peNDF_>1.18_ (particle size >1.18 mm) contents of 32.97, 29.93, 28.14, 26.48, and 24.75% were obtained by chopping or crusher crushing the forage into the following lengths: long (7 cm), medium (4 cm), short (1 cm), fine (5 mm sieve), very fine (1 mm sieve).

Different letters^a−c^ denote p < 0.05.

### Correlation analysis

**Figure 5:**
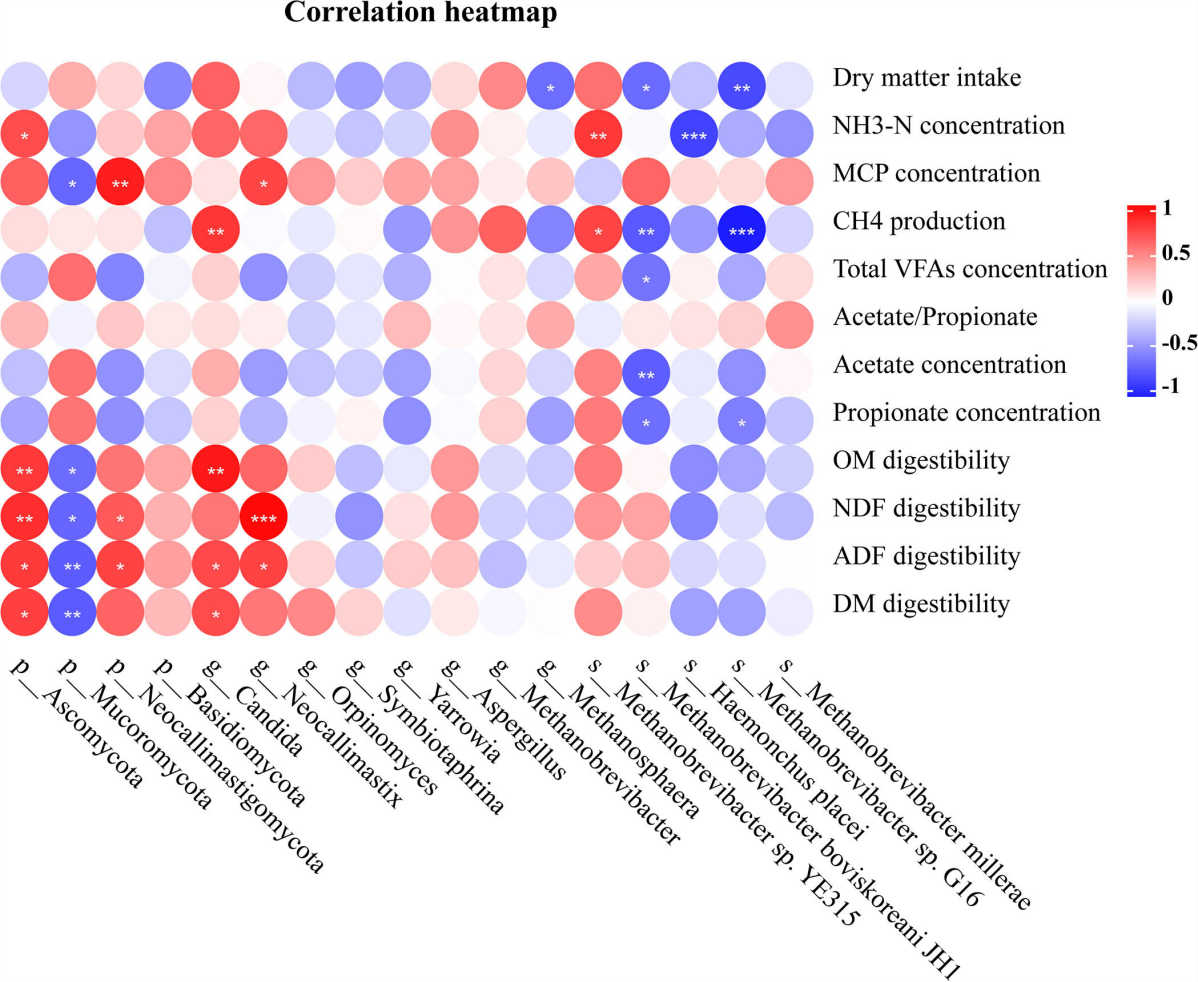
The correlation of dry matter intake, rumen fermentation parameters and nutrient digestibility with microbial abundance. The colors indicate positive (red, closer to 0.81) or negative (blue, closer to −0.90) correlations.

Correlations between microbiome composition and DM intake, digestibility, and rumen fermentation parameters are shown in Figure 5. The DM intake was negatively associated with the relative abundance of *s_Methanobrevibacter sp. G16, s_M. boviskoreani JH1* and *g_Methanosphaera* (r < −0.6 and *p* < 0.05). The concentration of NH_3_-N was negatively associated with the relative abundance of *s_Haemonchus placei* (r < −0.7 and *p* < 0.05), and it was also positively correlated with the relative abundance of *s_Methanobrevibacter sp. YE315* and p_Ascomycota (r > 0.5 and *p* < 0.05). The concentration of MCP was negatively associated with the relative abundance of p_Mucoromycota (r < −0.6 and *p* < 0.01), and it was also positively correlated with the relative abundance of p_Neocallimastigomycota and *g_Neocallimastix* (r > 0.6 and *p* < 0.05). The daily CH_4_ production was negatively associated with the relative abundance of *s_M. boviskoreani JH1* and *s_Methanobrevibacter sp. G16* (r < −0.6 and *p* < 0.01), and it was also positively correlated with the relative abundance of *g_Candida* and *s_Methanobrevibacter sp. YE315* (r > 0.6 and *p* < 0.05). The concentration of total VFAs was negatively associated with the species *s_M. boviskoreani JH1* (r < −0.5 and *p* < 0.05). The acetate concentration was negatively associated with the relative abundance of *s_M. boviskoreani JH1* (r < −0.6 and *p* < 0.05). The propionate concentration was negatively associated with the relative abundance of *s_M. boviskoreani JH1* and *s_Methanobrevibacter sp. G16* (r < −0.5 and *p* < 0.05). The digestibility of OM was negatively associated with the relative abundance of p_Mucoromycota (r < −0.6 and *p* < 0.05), while positively associated with the relative abundance of g_Candida and p_Ascomycota (r > 0.6 and *p* < 0.05). The digestibility of NDF was negatively associated with the relative abundance of p_Mucoromycota (r < −0.6 and *p* < 0.05), while positively associated with the relative abundance of p_Ascomycota, p_Neocallimastigomycota, and *g_Neocallimastix* (r > 0.5 and *p* < 0.05). The digestibility of ADF was negatively associated with the relative abundance of p_Mucoromycota (r < −0.6 and *p* < 0.05), while positively associated with the relative abundance of p_Ascomycota, p_Neocallimastigomycota, *g_Neocallimastix*, and *g_Candida* (r > 0.5 and *p* < 0.05). The digestibility of DM was negatively associated with the relative abundance of p_Mucoromycota (r < −0.6 and *p* < 0.01), while positively associated with the relative abundance of p_Ascomycota and *g_Candida* (r > 0.5 and *p* < 0.05).

## Discussion

### Effects of dietary peNDF on apparent nutrient digestibility

Due to the high forage content in ruminant feed, optimizing forage particle size is a significant feeding strategy to improve forage utilization for ruminants (Tafaj et al., 2005). A suitable particle size would lead to rapid digestion and passage of feed in the rumen, with less stimulation of masticatory activity and rumen contraction (Mertens, 1997). Excessively small or fine particle size would result in a decrease in rumen pH, negatively affecting fiber digestion, feed intake, and feed efficiency (Tafaj et al., 2005). In our recently published study, the feed intake of goats increased first and then decreased with the decrease of dietary peNDF_>1.18_ content from 33.0 to 24.8% (678 vs. 704 vs. 737 vs. 831 vs. 702 g/day), and reached the maximum at the content of peNDF_>1.18_ was 26.5% (Xue et al., 2022). The same trend appeared in the apparent digestibility of nutrients in this study, with the highest and the lowest digestibility of DM, OM, CP, NDF, and ADF observed in the 28.14% and 24.75% peNDF_>1.18_ treatment. It may be that more feed particles smaller than 1.88 mm have a faster passage rate from the rumen to escape rumen fermentation (Poppi et al., 1980), making against the digestion of nutrients, especially for the fiber. Jang et al. (2017) fed three diets with peNDF_>1.18_ content of 16.22, 23.85, and 21.71%, and found that the total rumen retention and transit time of feed increased with the escalating peNDF_>1.18_ content in the diet, providing more time for the digestion of nutrients. In addition, the particle size of ruminant feed also affected rumination and chewing behavior (Ramirez Ramirez et al., 2016; Jiang et al., 2019), determining the amount of saliva produced and secreted. Saliva is a buffer for maintaining ruminal pH, and acetate to propionate ratio, providing a stable environment for the fermentability and proper growth of rumen microbes (Banakar et al., 2018). Longer forage particles would be reduced in size during the prolonged chewing process, so as to partially eliminate the influence of particle length (Yang and Beauchemin, 2006c). Since 1.18 mm is considered the critical length for retention of chyme granules in the rumen, stimulating chewing and rumination behavior (Poppi et al., 1980, 1981), the apparent digestibility is poorly different in goats-fed diets with long (7 cm), medium (4 cm), and short (1 cm) forages in this study. Wang et al. (2017) investigated the effect of four dietary peNDF_>1.18_ levels (from 22.7% to 28.4%) on the nutrient digestibility of heifers and found that the digestibility increased when dietary peNDF_>1.18_ level increased from 22.7 to 25.9%, which was consistent with the results of this study. However, other studies reported that increasing dietary peNDF content reduced the apparent digestibility of DM, OM, NDF, and ADF (Soita et al., 2002; Yang and Beauchemin, 2006a) or did not affect the apparent digestibility (Park et al., 2014). The discrepancy in these findings may be due to the different ways of changing the content of peNDF and the type of concentrates. Wang et al. (2017) used the same corn grain-based diet and cut different forage lengths to control the peNDF content of the diet. Other studies, however, used barley as the main concentrate (Yang and Beauchemin, 2006a) or no concentrate (Soita et al., 2002) in the diet or adjusted the mixing time of TMR to control the content of dietary peNDF (Park et al., 2014). Fermentability of concentrate is also one of the important factors affecting the digestion of ruminants (Bekele et al., 2010). Compared with corn, the higher fermentability of the grain may be another factor affecting the digestion of nutrition (Yang and Beauchemin, 2006b).

Oh et al. (2016) found that the nutrient digestibility linearly increased with the dietary peNDF_>1.18_ content in heifers, in which the peNDF_>1.18_ levels ranged from 27.50 to 30.36%. The wide range of the peNDF_>1.18_ gradient (24.75–32.97%) in this study was useful in depicting the locus of nutrient digestibility with peNDF_>1.18_ levels. Regression analysis indicated that the digestibility of all nutrients reached the peak at the dietary peNDF_>1.18_ level of 30% (Table 4). The nutrient digestibility was quadratically correlated to dietary peNDF_>1.18_ content, and the inflection point falls in the range from 28.14 to 30.0%. Combining the results from Wang et al. (2017) and Oh et al. (2016), we deduced that the nutrient digestibility of ruminants kept on increasing when dietary peNDF_>1.18_ levels increased from 22.7 to 30.36%. It is well known that the increase in the residence time of feed in the gastrointestinal tract would improve its digestive efficiency (Shaver et al., 1988; Kaske and Engelhardt, 1990). In addition, the residence time in the gastrointestinal tract of 1.00 mm particles was 22.7 h less than that of 10.00 mm particles (Kaske and Engelhardt, 1990). Combined with the results of nutrient digestibility in this study, proper control of forage length (5.00–10.00 mm) was beneficial to the utilization of nutrients in goats.

### Effects of dietary peNDF on characteristics of ruminal fermentation in goats

Rumen pH reflects the balance between the production and absorption of organic acids in the rumen of ruminants. Physically effective fiber is the fraction of feed that stimulates chewing activity and salivary secretion (Mertens, 1997), which is beneficial to neutralizing excess hydrogen ions and therefore helps prevent the reduction of rumen pH. Our results suggested that dietary peNDF_>1.18_ content has no effect on rumen pH and VFAs concentration in the rumen. This may be due to the stable non-fibrous carbohydrate content across the diets. Although the content of peNDF in the diet is an important factor in maintaining the stability of the rumen environment (Allen, 1997), the rumen pH is more affected by the proportion of non-fiber carbohydrates in the diet, because they are more easily degraded in the rumen (Nasrollahi et al., 2012). Moreover, this study only measured the rumen pH at the time of slaughter before morning feeding and did not observe the dynamic change of rumen pH, all of this may result that no significant difference was observed between the groups.

However, our study found that the concentrates of NH_3_-N and MCP in the rumen were affected by the dietary peNDF_>1.18_ content. As the length of the forage particles decreased, the residence and fermentation time of the chyme in the rumen were shortened (Kaske and Engelhardt, 1990), which may be the reason for the low concentration of NH_3_-N and MCP in very fine treatment (peNDF_>1.18_ = 24.75%). Rumen NH_3_-N is an important nitrogen source for rumen microbial growth and microbial protein synthesis (Pisulewski et al., 2010). Wang et al. (2017) found that the ammonia concentration increased significantly with the increase of particle size and dietary peNDF_>1.18_ content, even though the peNDF_>1.18_ content in the diet was low (22.7 to 28.4%). In this study, the NH_3_-N concentration went up at first and then dropped with the decrease of the dietary peNDF_>1.18_ content from 33.0 to 24.8%, and reached the maximum at the content of peNDF_>1.18_ was 28.14%. This result is similar to the apparent digestibility of nutrients, with a quadratic linear relationship between rumen NH_3_-N and dietary peNDF_>1.18_ content.

Methane is the end-product of the degradation of dietary carbohydrates into VFAs in the rumen (Pinares-Patiño et al., 2007; Hylemon et al., 2018). Earlier studies have reported that CH_4_ production was positively correlated with the rate of feed degradation *in vitro* (Wang et al., 2014; Molina-Botero et al., 2020). The methane production in the 24.5% peNDF_>1.18_ treatment was the lowest in this study, consistent with the results of nutrient digestibility, this reduction might be related to the decrease in feed fermentation rate in the rumen. Furthermore, the variation in daily CH_4_ production when cattle are fed *ad libitum* is largely due to the differences in feed intake and feed nutritive value (Johnson and Johnson, 1995). The intake and nutritional value of feed determine the fermentation substrate of rumen microorganisms. A recent meta-analysis demonstrated a strong positive correlation between daily CH_4_ production and DMI in finishing beef cattle (Smith et al., 2021). Hence, we found that the variation trend of daily CH_4_ production was consistent with the DMI when goats were fed diets with different levels of peNDF (Xue et al., 2022), while the daily CH_4_ production per kg DMI was not affected by the dietary peNDF content.

### Effects of dietary peNDF level on rumen archaea

Archaea only account for 2–4% of the total rumen microorganisms of ruminants, making it difficult to study the structure and composition of rumen archaea (Lin et al., 1997; Cruz et al., 2016). Furthermore, the strains identified by *in vitro* culture were short of representation due to the fact that these archaea are strictly anaerobic organisms (Paul et al., 2017). Much more identification efforts are awaited to describe the classification of major archaeal species (Paul et al., 2017). Nevertheless, ruminal archaea are responsible for converting hydrogen and carbon dioxide produced by other fermenting microorganisms to methane, thereby playing a non-negligible role in maintaining rumen metabolism and function (Patra et al., 2017). In the current study, all sequences were clustered into three genera with validly published names and an unknown genus, and nine species with validly published names and two unknown species. Moreover, we found that *Methanobacter* was the most dominant genus in goat rumen, which is consistent with the previously reported results in buffaloes (Kala et al., 2020), goats (Fliegerova et al., 2021), sheep (McLoughlin et al., 2020), and beef cattle (Paul et al., 2017). The most dominant archaeal species was *Methanobrevibacter sp. YE315* and its relative abundance were significantly affected by dietary peNDF content. Unfortunately, we have not found any reports describing the role of *Methanobrevibacter sp. YE315*. The correlation analysis showed that the daily production of CH_4_ was positively correlated with the relative abundance of *Methanobrevibacter sp. YE315*, while negatively correlated with the relative abundances of both *Methanobrevibacter sp. G16* and *M. boviskoreani JH1*. The relationship between methanogenesis and the abundance of methanogens in the rumen has not been unified in the literature. Previous studies suggested that methane production was related to specific methanogens species or to the overall abundance of methanogens (Wallace et al., 2014; Danielsson et al., 2017). However, other reports showed that there was no relationship between methane production and the abundance of methanogens (Shi et al., 2014; Casañas et al., 2015). The current study concluded that the low relative abundance of methanogens in the rumen compared to fungi and bacteria contributes to the low or no correlation with methane production (Janssen and Kirs, 2008).

### Effects of dietary peNDF level on rumen fungi

Fungi account for 5–20% of the total microbial mass in the rumen of ruminants (Rezaeian et al., 2004). Fungi produce a large, comprehensive array of enzymes necessary for the digestion of plant material, such as cellulases, xylanases, and other hydrolases (Solomon et al., 2016). Due to the diversity and high activity of these enzymes, rumen fungi play a major role in lignocellulose fermentation (Orpin and Joblin, 1997). An earlier study showed that feeding a high proportion of enriched diets resulted in a significant reduction or even complete disappearance of fungal abundance in the rumen of 80% of lambs, while the fungal structure was restored to stability after feeding Alfalfa hay (Fonty et al., 1987). Another study also showed that increasing the proportion of concentrate in the diet would reduce the total number of rumen fungi in sheep (Faichney et al., 1997). These studies indicated that the composition and structure of rumen fungi were affected by the content of forage (fiber) in the diet. Rumen fungi are closely related to the number of plant substrates in the rumen due to their properties of colonizing fiber granules (Gordon and Phillips, 1998). In addition, it has been reported that fungi play an indispensable role in the degradation of cellulose in the rumen because they can penetrate deeply into plant tissues that are usually inaccessible to bacteria (Bauchop and Mountfort, 1981), which has been confirmed in controlling fungal activity to change the degradation rate of plant cell walls (Lee et al., 2000). Neocalimastigales family members play an important role in degrading the feed materials that has complex structures and are rich in cellulose, enabling the production of hydrogen gas as a substrate for other rumen microorganisms (Boots et al., 2013). The proportion of concentrate in the diet increased from 50 to 90% separated Neocallismastigales assemblies (Boots et al., 2013). In this study, we found that the relative abundance of *f_Neocallimastigaceae|g_uncultured* was the highest in the 32.97% peNDF_>1.88_ treatment and the lowest in the 28.14% peNDF_>1.88_ treatment, indicating that the content of dietary peNDF affected its abundance. *Neocallimstixhas* was observed to be the dominant fungal genus in the rumen of most ruminants (Kittelmann et al., 2013), which is consistent with the results of the current study. *Neocallimastix* produces lower amounts of cellulolytic and xylanolytic enzymes than other fungi (e.g., *Piromyces*) (Teunissen et al., 1992). The content of peNDF in the diet affected the abundance of *Neocallimastix* in this study. Overall, diet plays an important role in maintaining fungal diversity in the rumen of ruminants. The anaerobic rumen fungal population in the rumen of cattle fed the same growing stage forages was reduced compared with those fed mature forages (Kostyukovsky et al., 1991). Notably, microbes that comprise a larger proportion of the abundance contribute significantly to the function of the rumen microbial ecosystem, and a small subset of microbes in the rumen community may have important, yet unrecognized, ecological functions (Singh et al., 2015).

### Effects of dietary peNDF level on microbial functions

In this study, we inferred the function of microorganisms in the rumen of goats by Tax4Fun and FUNGuild. Many genes from rumen microbiota are closely related to nutrient metabolism, and therefore can be used to predict the function of microbial activity in nutrient metabolism (Noecker et al., 2016). The metabolic genes of archaea in the 24.75% peNDF_>1.18_ treatment is low, suggesting that low peNDF may not be beneficial to the metabolic activities of archaea. The production of methane is also associated with feed intake and rumen passage rate in addition to the abundance or activity of methanogens (Goopy et al., 2014; Fagundes et al., 2020). Both feed intake and rumen passage rate may affect the growth of rumen microbes (including methanogens) because they are related to the microbial energy flux and microbial production time (Satter, 1986; Martínez et al., 2009). The lower DM intake in the 32.97% peNDF_>1.88_ and 24.75% peNDF_>1.88_ treatments could result in a decrease in the relative abundance of *Methanobrevibacter sp. YE315*. The very fine forage particles in the 24.75% peNDF_>1.88_ treatment could increase the rumen passage rate, thus decreasing the relative abundance of *Methanobrevibacter sp. YE315*. The functional prediction value of carbohydrate metabolism was observed to be lower in the 32.97% peNDF_>1.88_ and 24.75% peNDF_>1.88_ treatments. These results were consistent with the daily production of CH_4_. On the other hand, archaea in rumen interact with fungi and bacteria (Patra et al., 2017). Although little is known about archaea related to fungi, rumen bacteria and archaea have been confirmed to interact through interspecific hydrogen transfer (Patra et al., 2017). A clear link between rumen fungal abundance and methane production has been demonstrated by experiments *in vitro* (Newbold et al., 1995; Qin et al., 2012) and *in vivo* (Schönhusen et al., 2003), implying that there was an association between fungi and archaea.

Based on the mode of nutrient acquisition (trophic mode), fungi can be classified into three main groups (Tedersoo et al., 2014): saprotroph, nutrients derived from degradation of dying host cells; symbiotroph, nutrients derived from exchanging resources with host cells; pathotroph, nutrients derived from harming host cells. Further, in these trophic modes, a total of 12 categories were included as guilds: animal pathogens, arbuscular mycorrhizal fungi, ectomycorrhizal fungi, ericoidmycorrhizal fungi, foliar endophytes, lichenicolous fungi, liche-nized fungi, mycoparasites, plant pathogens, undefined root en-dophytes, undefined saprotrophs, and wood saprotrophs. We found that the guild OUT richness of pathotroph was the lowest in the 28.14% peNDF_>1.88_ treatment while the highest in the 26.48% peNDF_>1.88_ treatment, indicating that the dietary peNDF level of 26.48% was unfavorable to the rumen health of the host.

## Conclusion

To the best of our knowledge, this report is the first study to investigate the effect of dietary peNDF levels on rumen fungal diversity in goats. In this study, we reduced the content of peNDF_>1.18_ in diets by reducing the chop length of forages, and found that the community structures of rumen archaea and fungi varied with the levels of dietary peNDF_>1.18_. Without changing the composition of the diet, appropriately reducing the levels of peNDF_>1.18_ in the diet contributed to higher nutrient digestibility in goats, whereas excessive crushing resulted in negative effects. The apparent digestibility of nutrients was highest in the 28.14% PeNDF_>1.18_ treatment, indicating that the nutrient utilization was optimal at this level of peNDF_>1.18_. Therefore, we recommend that the content of peNDF_>1.18_ in goat diets should be 28.14% (roughage chopped to 1 cm) to get higher usage of feed. Moreover, whether the level of peNDF in the diets affected the production performance of ruminants, such as milk yield, growth performance, and meat quality, needs to be further studied.

## Data availability statement

The datasets generated for this study can be found in online repositories. The names of the repository/repositories and accession number(s) can be found at: https://www.ncbi.nlm.nih.gov/, PRJNA865508.

## Ethics statement

The animal study was reviewed and approved by the Animal Ethical and Welfare Committee of Animal Nutrition Institute, Sichuan Agricultural University.

## Author contributions

JZ and BeX wrote the original manuscript, performed data statistical analysis, and produced figures and tables. BeX and MW performed the experiments. AH and YW provided great help in data analysis. JZ, BaX, and QH participated in the design of the trial. SY, ZW, BaX, LW, and QP revised the manuscript. All authors have read and approved the final manuscript.

## Funding

This research was funded by China Agriculture Research System of MOF and MARA (CARS-39).

## Conflict of interest

The authors declare that the research was conducted in the absence of any commercial or financial relationships that could be construed as a potential conflict of interest.

## Publisher's note

All claims expressed in this article are solely those of the authors and do not necessarily represent those of their affiliated organizations, or those of the publisher, the editors and the reviewers. Any product that may be evaluated in this article, or claim that may be made by its manufacturer, is not guaranteed or endorsed by the publisher.

## References

[B1] Adesogan A. Arriola K. Jiang Y. Oyebade A. Paula E. Pech-Cervantes A.. (2019). Symposium review: technologies for improving fiber utilization. J. Dairy Sci. 102, 5726–5755. 10.3168/jds.2018-15334 30928262

[B2] Aguilera J. Prieto C. (1986). Description and function of an open-circuit respiration plant for pigs and small ruminants and the techniques used to measure energy metabolism. Arch. Anim. Nutr. 36,1009–1018. 10.1080/17450398609429522 2949721

[B3] Allen M. S. (1997). Relationship between fermentation acid production in the rumen and the requirement for physically effective fiber. J.Dairy Sci. 80, 1447–1462. 10.3168/jds.S0022-0302(97)76074-0 9241607

[B4] Almeida F. Stein H. (2010). Performance and phosphorus balance of pigs fed diets formulated on the basis of values for standardized total tract digestibility of phosphorus. J. Anim. Sci. 88, 2968–2977. 10.2527/jas.2009-2285 20495131

[B5] Banakar P. Anand Kumar N. Shashank C. (2018). Physically effective fibre in ruminant nutrition: a review. J. Pharmacogn. Phytochem. 7, 303–308

[B6] Bargo F. Muller L. Delahoy J. Cassidy T. (2002). Performance of high producing dairy cows with three different feeding systems combining pasture and total mixed rations. J. Dairy Sci. 85, 2948–2963. 10.3168/jds.S0022-0302(02)74381-6 12487461

[B7] Bauchop T. Mountfort D. O. (1981). Cellulose fermentation by a rumen anaerobic fungus in both the absence and the presence of rumen methanogens. Appl. Environ. Microb. 42, 1103–1110. 10.1007/BF00443249 16345902 PMC244160

[B8] Bekele A. Z. Koike S. Kobayashi Y. (2010). Genetic diversity and diet specificity of ruminal Prevotella revealed by 16S rRNA gene-based analysis. FEMS Microbiol. Lett. 305, 49–57. 10.1111/j.1574-6968.2010.01911.x 20158525

[B9] Berthiaume R. Benchaar C. Chaves A. Tremblay G. Castonguay Y. Bertrand A.. (2010). Effects of nonstructural carbohydrate concentration in alfalfa on fermentation and microbial protein synthesis in continuous culture. J. Dairy Sci. 93, 693–700. 10.3168/jds.2009-2399 20105540

[B10] Billenkamp F. Schnabel K. Hüther L. Frahm J. von Soosten D. Meyer U.. (2021). No hints at glyphosate-induced ruminal dysbiosis in cows. NPJ Biofilms. Microbi. 7, 1–13. 10.1038/s41522-021-00198-4 33767196 PMC7994389

[B11] Boots B. Lillis L. Clipson N. Petrie K. Kenny D. Boland T.. (2013). Responses of anaerobic rumen fungal diversity (phylum N eocallimastigomycota) to changes in bovine diet. J. Appl. Microbiol. 114, 626–635. 10.1111/jam.12067 23163953

[B12] Cao Y. Gao Y. Xu M. Liu N. Zhao X. Liu C.. (2013). Effect of ADL to aNDF ratio and ryegrass particle length on chewing, ruminal fermentation, and in situ degradability in goats. Anim. Feed Sci. Tech. 186, 112–119. 10.1016/j.anifeedsci.2013.08.010

[B13] Cao Y. Wang D. Wang L. Wei X. Li X. Cai C.. (2021). Physically effective neutral detergent fiber improves chewing activity, rumen fermentation, plasma metabolites, and milk production in lactating dairy cows fed a high-concentrate diet. J. Dairy Sci. 104, 5631–5642. 10.3168/jds.2020-19012 33663818

[B14] Casañas M. A. Rangkasenee N. Krattenmacher N. Thaller G. Metges C. Kuhla B. J. J.. (2015). Methyl-coenzyme M reductase A as an indicator to estimate methane production from dairy cows. J. Dairy Sci. 98, 4074–4083. 10.3168/jds.2015-9310 25841964

[B15] Cruz G. D. Millen D. D. Rigueiro A. L. N. (2016). Rumen models, in Rumenology, eds Millen D. De Beni Arrigoni M. Lauritano Pacheco R. (Cham: Springer). 10.1007/978-3-319-30533-2_10

[B16] Danielsson R. Dicksved J. Sun L. Gonda H. Müller B. Schnürer A.. (2017). Methane production in dairy cows correlates with rumen methanogenic and bacterial community structure. Front. Microbiol. 8, 226. 10.3389/fmicb.2017.00226 28261182 PMC5313486

[B17] de Paula Carlis M. S. Sturion T. U. da Silva A. L. A. Eckermann N. R. Polizel D. M. de Assis R. G.. (2021). Whole corn grain-based diet and levels of physically effective neutral detergent fiber from forage (pefNDF) for feedlot lambs: Digestibility, ruminal fermentation, nitrogen balance and ruminal pH. Small Ruminant. Res. 205, 106567. 10.1016/j.smallrumres.2021.106567

[B18] Edgar R. C. (2013). UPARSE: highly accurate OTU sequences from microbial amplicon reads. Nat. Methods 10, 996–998. 10.1038/nmeth.2604 23955772

[B19] Fagundes G. M. Benetel G. Welter K. C. Melo F. A. Muir J. P. Carriero M. M.. (2020). Tannin as a natural rumen modifier to control methanogenesis in beef cattle in tropical systems: friend or foe to biogas energy production? Res. Vet. Sci. 132, 88–96. 10.1016/j.rvsc.2020.05.010 32540589

[B20] Faichney G. Poncet C. Lassalas B. Jouany J. Millet L. Dor,é J.. (1997). Effect of concentrates in a hay diet on the contribution of anaerobic fungi, protozoa and bacteria to nitrogen in rumen and duodenal digesta in sheep. Anim. Feed Sci. Tech. 64, 193–213. 10.1016/S0377-8401(96)01059-0

[B21] Fliegerova K. O. Podmirseg S. M. Vinzelj J. Grilli D. J. Kvasnov,á S Schierov,á D.. (2021). The effect of a high-grain diet on the rumen microbiome of goats with a special focus on anaerobic fungi. Microorganisms 9, 157. 10.3390/microorganisms9010157 33445538 PMC7827659

[B22] Fonty G. Gouet P. Jouany J.-P. Senaud J. (1987). Establishment of the microflora and anaerobic fungi in the rumen of lambs. Microbiology 133, 1835–1843. 10.1099/00221287-133-7-1835

[B23] Goopy J. P. Donaldson A. Hegarty R. Vercoe P. E. Haynes F. Barnett M.. (2014). Low-methane yield sheep have smaller rumens and shorter rumen retention time. Br. J. Nutr. 111, 578–585. 10.1017/S0007114513002936 24103253

[B24] Gordon G. L. Phillips M. W. (1998). The role of anaerobic gut fungi in ruminants. Nutr. Res. Rev. 11, 133–168. 10.1079/nrr19980009 19087463

[B25] Hylemon P. B. Harris S. C. Ridlon J. M. (2018). Metabolism of hydrogen gases and bile acids in the gut microbiome. FEBS Lett. 592, 2070–2082. 10.1002/1873-3468.13064 29683480

[B26] International A. (1990). Official methods of analysis. Gaithersburg, Washington, DC, USA: Aoac Intl.

[B27] Jang S. Y. Kim E. K. Park J. H. Oh M. R. Tang Y. J. Ding Y. L.. (2017). Effects of physically effective neutral detergent fiber content on dry matter intake, digestibility, and chewing activity in Korean native goats (Capra hircus coreanae) fed with total mixed ration. Asian. Austral. J. Anim. 30, 1405. 10.5713/ajas.16.0868 28423870 PMC5582324

[B28] Janssen P. H. Kirs M. (2008). Structure of the archaeal community of the rumen. Appl. Environ. Microbiol. 74, 3619–3625. 10.1128/AEM.02812-07 18424540 PMC2446570

[B29] Jiang F. G. Lin X. Y. Yan Z. G. Hu Z. Y. Wang Y. Wang Z. H. (2019). Effects of forage source and particle size on chewing activity, ruminal pH, and saliva secretion in lactating Holstein cows. Anim. Sci. J. 90, 382–392. 10.1111/asj.13153 30661262

[B30] Johnson K. A. Johnson D. E. (1995). Methane emissions from cattle. J. Anim. Sci. 73, 2483–2492. 10.2527/1995.7382483x 8567486

[B31] Kaeokliang O. Kawashima T. Narmseelee R. Butcha P. Sunato S. Thinowong A.. (2019). Effects of physically effective fiber in diets based on rice straw and cassava pulp on chewing activity, ruminal fermentation, milk production, and digestibility in dairy cows. Anim. Sci. J. 90, 1193–1199. 10.1111/asj.13271 31310041

[B32] Kala A. Kamra D. Agarwal N. Chaudhary L. Joshi C. (2020). Insights into metatranscriptome, and CAZymes of buffalo rumen supplemented with blend of essential oils. Indian J. Microbiol. 60, 485–493. 10.1007/s12088-020-00894-3 33087998 PMC7539254

[B33] Kaske M. Engelhardt W. (1990). The effect of size and density on mean retention time of particles in the gastrointestinal tract of sheep. Brit. J. Nutr. 63, 457–465. 10.1079/BJN19900133 2383525

[B34] Kittelmann S. Seedorf H. Walters W. A. Clemente J. C. Knight R. Gordon J. I.. (2013). Simultaneous amplicon sequencing to explore co-occurrence patterns of bacterial, archaeal and eukaryotic microorganisms in rumen microbial communities. PloS ONE. 8, e47879. 10.1371/journal.pone.0047879 23408926 PMC3568148

[B35] Kõljalg U. Nilsson R. H. Abarenkov K. Tedersoo L. Taylor A. F. Bahram M.. (2013). Towards a unified paradigm for sequence-based identification of fungi. Mol. Ecol. 22, 5271–5277. 10.1111/mec.12481 24112409

[B36] Kostyukovsky V. A. Okunev O. N. Tarakanov B. V. (1991). Description of two anaerobic fungal strains from the bovine rumen and influence of diet on the fungal population in vivo. Microbiology. 137, 1759–1764. 10.1099/00221287-137-7-1759 1955864

[B37] Lee S. Ha J. Cheng K.-J. (2000). Relative contributions of bacteria, protozoa, and fungi to in vitro degradation of orchard grass cell walls and their interactions. Appl. Environ. Microbiol. 66, 3807–3813. 10.1128/AEM.66.9.3807-3813.2000 10966394 PMC92224

[B38] Li C. Beauchemin K. A. Yang W. (2020). Feeding diets varying in forage proportion and particle length to lactating dairy cows: I. effects on ruminal pH and fermentation, microbial protein synthesis, digestibility, and milk production. J. Dairy Sci. 103, 4340–4354. 10.3168/jds.2019-17606 32197848

[B39] Li F. Guan L. L. (2017). Metatranscriptomic profiling reveals linkages between the active rumen microbiome and feed efficiency in beef cattle. Appl. Environ. Microb. 83, e00061–e00017. 10.1128/AEM.00061-17 28235871 PMC5394315

[B40] Li F. Li Z. Li S. d Ferguson J. Cao Y. Yao J.. (2014). Effect of dietary physically effective fiber on ruminal fermentation and the fatty acid profile of milk in dairy goats. J. Dairy Sci. 97, 2281–2290. 10.3168/jds.2013-6895 24508430

[B41] Lin C. Raskin L. Stahl D. A. (1997). Microbial community structure in gastrointestinal tracts of domestic animals: comparative analyses using rRNA-targeted oligonucleotide probes. FEMS Microbio. Ecol. 22, 281–294. 10.1016/S0168-6496(97)00002-0

[B42] Martínez M. Ranilla M. Ramos S. Tejido M. Carro M. (2009). Effects of dilution rate and retention time of concentrate on efficiency of microbial growth, methane production, and ruminal fermentation in Rusitec fermenters. J. Dairy Sci. 92, 3930–3938. 10.3168/jds.2008-1975 19620676

[B43] Maulfair D. Fustini M. Heinrichs A. (2011). Effect of varying total mixed ration particle size on rumen digesta and fecal particle size and digestibility in lactating dairy cows. J. Dairy Sci. 94, 3527–3536. 10.3168/jds.2010-3718 21700040

[B44] McLoughlin S. Spillane C. Claffey N. Smith P. E. O'Rourke T. Diskin M. G.. (2020). Rumen microbiome composition is altered in sheep divergent in feed efficiency. Front. Microbiol. 11, 1981. 10.3389/fmicb.2020.01981 32983009 PMC7477290

[B45] Mertens D. (1997). Creating a system for meeting the fiber requirements of dairy cows. J. Dairy Sci. 80, 1463–1481. 10.3168/jds.S0022-0302(97)76075-2 9241608

[B46] Molavian M. Ghorbani G. Rafiee H. Beauchemin K. (2020). Substitution of wheat straw with sugarcane bagasse in low-forage diets fed to mid-lactation dairy cows: Milk production, digestibility, and chewing behavior. J. Dairy Sci. 103, 8034–8047. 10.3168/jds.2020-18499 32684450

[B47] Molina-Botero I. C. Mazabel J. Arceo-Castillo J. Urrea-Benítez J. L. Olivera-Castillo L. Barahona-Rosales R.. (2020). Effect of the addition of Enterolobium cyclocarpum pods and Gliricidia sepium forage to Brachiaria brizantha on dry matter degradation, volatile fatty acid concentration, and in vitro methane production. Trop. Anim. Health Pro. 52, 2787–2798. 10.1007/s11250-020-02324-4 32647965

[B48] Nasrollahi S. Khorvash M. Ghorbani G. Teimouri-Yansari A. Zali A. Zebeli Q. (2012). Grain source and marginal changes in forage particle size modulate digestive processes and nutrient intake of dairy cows. Animal 6, 1237–1245. 10.1017/S1751731112000122 23217227

[B49] Newbold C. Lassalas B. Jouany J. (1995). The importance of methanogens associated with ciliate protozoa in ruminal methane production in vitro. Lett. Appl. Microbiol. 21, 230–234. 10.1111/j.1472-765x.1995.tb01048.x 7576513

[B50] Noecker C. Eng A. Srinivasan S. Theriot C. M. Young V. B. Jansson J. K.. (2016). Metabolic model-based integration of microbiome taxonomic and metabolomic profiles elucidates mechanistic links between ecological and metabolic variation. MSystems 1, e00013–00015. 10.1128/mSystems.00013-15 27239563 PMC4883586

[B51] NRC. (2007). Nutrient requirements of small ruminants: sheep, goats, cervids, and new world camelids. The National Academies Press, Washington D.C. USA. 10.17226/11654

[B52] Oh M. R. Hong H. Li H. L. Jeon B. T. Choi C. H. Ding Y. L.. (2016). Effects of Physically Effective Neutral Detergent Fiber Content on Intake, Digestibility, and Chewing Activity in Fattening Heifer Fed Total Mixed Ration. Asian. Austral. J. Anim. 29, 1719. 10.5713/ajas.16.0344 27488845 PMC5088419

[B53] Orpin C. Joblin K. (1997). The rumen anaerobic fungi, in The rumen microbial ecosystem. p. 140–195. 10.1016/0378-1097(87)90045-0

[B54] Park J. H. Kim K. H. Park P. J. Jeon B. T. Oh M. R. Jang S. Y.. (2014). Effects of physically effective neutral detergent fibre content on dry-matter intake, digestibility and chewing activity in beef cattle fed total mixed ration. Anim. Prod. Sci. 55, 166–169. 10.1071/AN14241

[B55] Patra A. Park T. Kim M. Yu Z. (2017). Rumen methanogens and mitigation of methane emission by anti-methanogenic compounds and substances. J. Anim. Sci. Biotechno. 8, 1–18. 10.1186/s40104-017-0145-9 28149512 PMC5270371

[B56] Paul S. S. Dey A. Baro D. Punia B. S. (2017). Comparative community structure of archaea in rumen of buffaloes and cattle. J. Sci. Food Agr. 97, 3284–3293. 10.1002/jsfa.8177 27976411

[B57] Pinares-Patiño C. Waghorn G. Machmüller A. Vlaming B. Molano G. Cavanagh A.. (2007). Methane emissions and digestive physiology of non-lactating dairy cows fed pasture forage. Can. J. Anim. Sci. 87, 601–613. 10.4141/CJAS06023

[B58] Pisulewski P. M. Okorie A. U. Buttery P. J. Haresign W. Lewis D. (2010). Ammonia concentration and protein synthesis in the rumen. P. Nut. Soc. 32, 759–766. 10.1002/jsfa.2740320803 7289584

[B59] Poppi D. Minson D. Ternouth J. (1981). Studies of cattle and sheep eating leaf and stem fractions of grasses. 1. The voluntary intake, digestibility and retention time in the reticulo-rumen. Aust. J. Agr. Res. 32, 99–108. 10.1071/ar9810099

[B60] Poppi D. Norton B. Minson D. Hendricksen R. (1980). The validity of the critical size theory for particles leaving the rumen. J. Agri. Sci. 94, 275–280. 10.1017/s0021859600028859

[B61] Qin W. Li C. Kim J. Ju J. Song M.-K. (2012). Effects of defaunation on fermentation characteristics and methane production by rumen microbes in vitro when incubated with starchy feed sources. Asian-Australas. J. Anim. Sci. 25, 1381. 10.5713/ajas.2012.12240 25049493 PMC4093010

[B62] Quast C. Pruesse E. Yilmaz P. Gerken J. Schweer T. Yarza P.. (2012). The SILVA ribosomal RNA gene database project: improved data processing and web-based tools. Nucleic Acids Res. 41, D590–D596. 10.1093/nar/gks1219 23193283 PMC3531112

[B63] Ramirez Ramirez H. Harvatine K. Kononoff P. (2016). Short communication: Forage particle size and fat intake affect rumen passage, the fatty acid profile of milk, and milk fat production in dairy cows consuming dried distillers grains with solubles. J. Dairy Sci. 99, 392–8. 10.3168/jds.2015-10006 26547654

[B64] Rezaeian M. Beakes G. W. Parker D. S. (2004). Distribution and estimation of anaerobic zoosporic fungi along the digestive tracts of sheep. Nucleic Acids Res. 108, 1227–1233. 10.1017/S0953756204000929 15535073

[B65] Satter L. (1986). Protein and fiber digestion, passage and utilization in lactating cows. J. Dairy Sci. 69, 2734–2749. 10.3168/jds.s0022-0302(87)80027-9 3805454

[B66] Schönhusen U. Zitnan R. Kuhla S. Jentsch W. Derno M. Voigt J. (2003). Effects of protozoa on methane production in rumen and hindgut of calves around time of weaning. Arch. Anim. Nutr. 57, 279–295. 10.1080/00039420310001594423 14533867

[B67] Shaver R. Nytes A. Satter L. Jorgensen N. (1988). Influence of feed intake, forage physical form, and forage fiber content on particle size of masticated forage, ruminal digesta, and feces of dairy cows. J. Dairy Sci. 71, 1566–1572. 10.3168/jds.S0022-0302(88)79720-9 2841366

[B68] Shi W. Moon C. D. Leahy S. C. Kang D. Froula J. Kittelmann S.. (2014). Methane yield phenotypes linked to differential gene expression in the sheep rumen microbiome. Genome Res. 24, 1517–1525. 10.1101/gr.168245.113 24907284 PMC4158751

[B69] Singh K. Jisha T. Reddy B. Parmar N. Patel A. Patel A.. (2015). Microbial profiles of liquid and solid fraction associated biomaterial in buffalo rumen fed green and dry roughage diets by tagged 16S rRNA gene pyrosequencing. Mol. Biol. Rep. 42, 95–103. 10.1007/s11033-014-3746-9 25249226

[B70] Smith P. E. Waters S. M. Kenny D. A. Kirwan S. F. Conroy S. Kelly A. K. (2021). Effect of divergence in residual methane emissions on feed intake and efficiency, growth and carcass performance, and indices of rumen fermentation and methane emissions in finishing beef cattle. J. Anim. Sci. 99, skab275. 10.1093/jas/skab275 34598276 PMC8598385

[B71] Soita H. Christensen D. McKinnon J. Mustafa A. (2002). Effects of barley silage of different theoretical cut length on digestion kinetics in ruminants. Can. J. Anim. Sci. 82, 207–213. 10.4141/A01-064

[B72] Solomon K. V. Haitjema C. H. Henske J. K. Gilmore S. P. Borges-Rivera D. Lipzen A.. (2016). Early-branching gut fungi possess a large, comprehensive array of biomass-degrading enzymes. Science. 351, 1192–1195. 10.1126/science.aad1431 26912365 PMC5098331

[B73] Tafaj M. Kolaneci V. Junck B. Maulbetsch A. Steingass H. Drochner W. (2005). Influence of fiber content and concentrate level on chewing activity, ruminal digestion, digesta passage rate and nutrient digestibility in dairy cows in late lactation. Asian. Austral. J. Anim. 18, 1116–1124. 10.1080/17450390500217082 16320816

[B74] Tedersoo L. Bahram M. Põlme S. Kõljalg U. Yorou N. S. Wijesundera R.. (2014). Global diversity and geography of soil fungi. Science. 346, 1256688. 10.1126/science.1256688 25430773

[B75] Teske A. Sørensen K. B. (2008). Uncultured archaea in deep marine subsurface sediments: have we caught them all? ISME. J. 2, 3–18. 10.1038/ismej.2007.90 18180743

[B76] Teunissen M. J. de Kort G. V. den Camp H. J. O. Huis J. H. (1992). Production of cellulolytic and xylanolytic enzymes during growth of the anaerobic fungus Piromyces sp. on different substrates. Microbiology. 138, 1657–1664. 10.1099/00221287-138-8-1657 1527505

[B77] Toju H. Kishida O. Katayama N. Takagi K. (2016). Networks depicting the fine-scale co-occurrences of fungi in soil horizons. PloS ONE. 11, e0165987. 10.1371/journal.pone.0165987 27861486 PMC5115672

[B78] Toju H. Tanabe A. S. Yamamoto S. Sato H. (2012). High-coverage ITS primers for the DNA-based identification of ascomycetes and basidiomycetes in environmental samples. PloS ONE. 7, e40863. 10.1371/journal.pone.0040863 22808280 PMC3395698

[B79] Van Soest P. (1963). A rapid method for the determination of fiber and lignin. J. Assoc. Off. Anal. Chem. 46, 829–835.

[B80] Wallace R. J. Rooke J. A. Duthie C.-A. Hyslop J. J. Ross D. W. McKain N.. (2014). Archaeal abundance in post-mortem ruminal digesta may help predict methane emissions from beef cattle. Sci. Rep. 4, 1–8. 10.1038/srep05892 25081098 PMC5376199

[B81] Wang H. Chen Q. Chen L. Ge R. Wang M. Yu L.. (2017). Effects of dietary physically effective neutral detergent fiber content on the feeding behavior, digestibility, and growth of 8-to 10-month-old Holstein replacement heifers. J. Dairy Sci. 100, 1161–1169. 10.3168/jds.2016-10924 27988115

[B82] Wang M. Sun X. Janssen P. Tang S. Tan Z. (2014). Responses of methane production and fermentation pathways to the increased dissolved hydrogen concentration generated by eight substrates in in vitro ruminal cultures. Anim. Feed Sci. Tech. 194, 1–11. 10.1016/j.anifeedsci.2014.04.012

[B83] Weatherburn M. (1967). Phenol-hypochlorite reaction for determination of ammonia. Anal. Chem. 39, 971–974. 10.1021/ac60252a045

[B84] Xue B. Wu M. Yue S. Hu A. Li X. Hong Q.. (2022). Changes in Rumen Bacterial Community Induced by the Dietary Physically Effective Neutral Detergent Fiber Levels in Goat Diets. Front. Microbiol. 13, 820509–820509. 10.3389/fmicb.2022.820509 35479630 PMC9035740

[B85] Yang W. Beauchemin K. (2005). Effects of physically effective fiber on digestion and milk production by dairy cows fed diets based on corn silage. J. Dairy Sci. 88, 1090–1098. 10.3168/jds.S0022-0302(05)72776-4 15738243

[B86] Yang W. Beauchemin K. (2006a). Increasing the physically effective fiber content of dairy cow diets may lower efficiency of feed use. J. Dairy Sci. 89, 2694–2704. 10.3168/JDS.S0022-0302(06)72345-1 16772588

[B87] Yang W. Beauchemin K. (2006b). Effects of physically effective fiber on chewing activity and ruminal pH of dairy cows fed diets based on barley silage. J. Dairy Sci. 89, 217–228. 10.3168/jds.S0022-0302(06)72086-0 16357285

[B88] Yang W. Beauchemin K. (2006c). Physically effective fiber: method of determination and effects on chewing, ruminal acidosis, and digestion by dairy cows. J. Dairy Sci. 89, 2618–2633. 10.3168/jds.S0022-0302(06)72339-6 16772582

[B89] Yang W. Beauchemin K. (2007). Altering physically effective fiber intake through forage proportion and particle length: Chewing and ruminal pH. J. Dairy Sci. 90, 2826–2838. 10.3168/jds.2007-0032 17517723

[B90] Yildirim E. Ilina L. Laptev G. Filippova V. Brazhnik E. Dunyashev T.. (2021). The structure and functional profile of ruminal microbiota in young and adult reindeers (Rangifer tarandus) consuming natural winter-spring and summer-autumn seasonal diets. PeerJ 9, e12389. 10.7717/peerj.12389 34900412 PMC8627130

[B91] Zebeli Q. Aschenbach J. Tafaj M. Boguhn J. Ametaj B. Drochner W. (2012). Invited review: Role of physically effective fiber and estimation of dietary fiber adequacy in high-producing dairy cattle. J. Dairy Sci. 95, 1041–1056. 10.3168/jds.2011-4421 22365188

[B92] Zhao X. Zhang T. Xu M. Yao J. (2011). Effects of physically effective fiber on chewing activity, ruminal fermentation, and digestibility in goats. J. Anim. Sci. 89, 501–509. 10.2527/jas.2010-3013 20935139

