# Supplementary material for: Effect of dietary peNDF levels on digestibility and rumen fermentation, and microbial community in growing goats
Source: Front Microbiol. 2022 Aug 25;13:950587. doi: 10.3389/fmicb.2022.950587 (PMC9453810; doi:10.3389/fmicb.2022.950587)
Supplement: Supplementary file 1 [file Presentation_1.pdf]

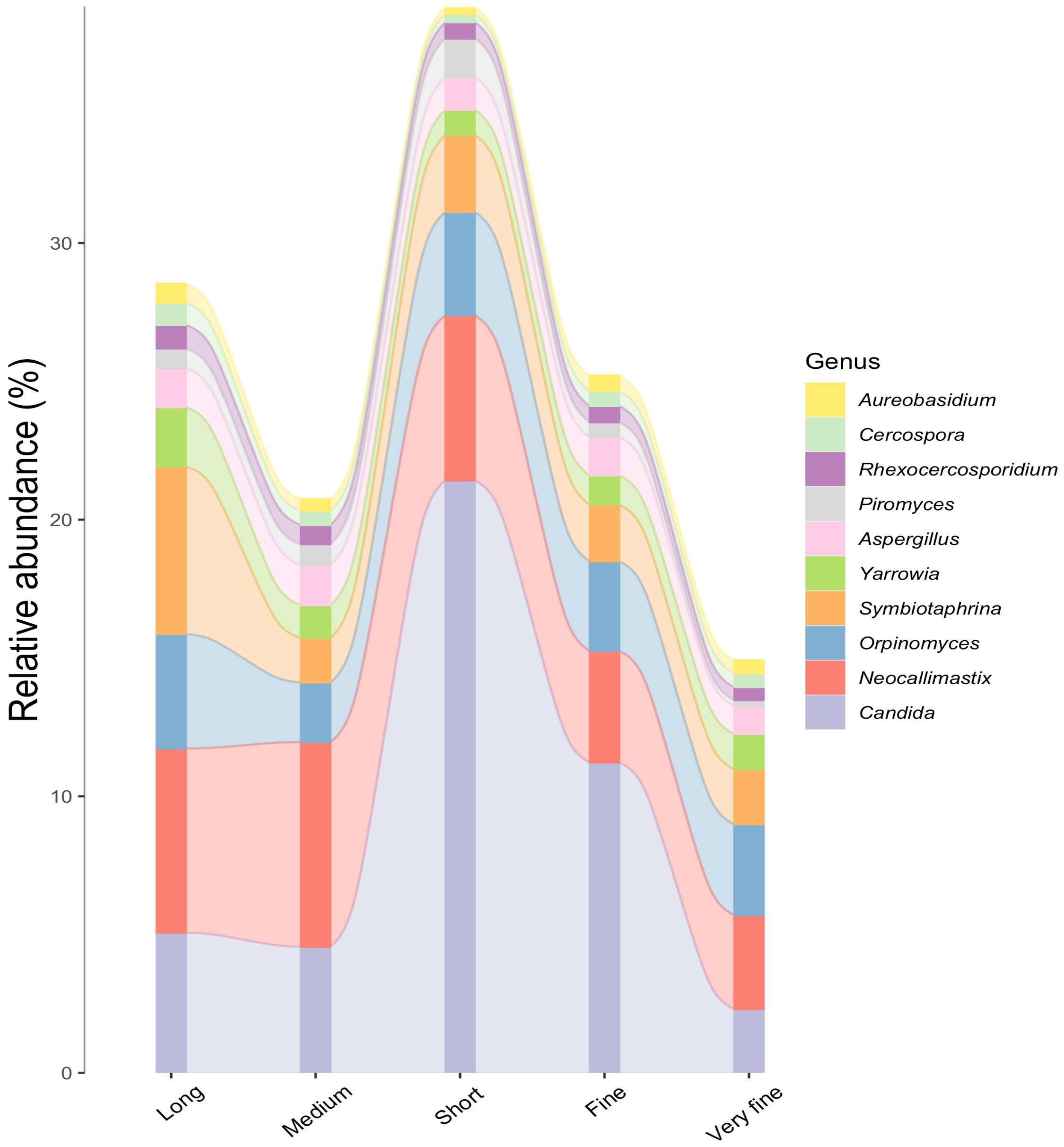

Figure S1. The sankeyplot of the relative abundance of fungi at genus level. Long: 32.97% peNDF>1.18 treatment; Medium: 29.93% peNDF>1.18 treatment; Short: 28.14% peNDF>1.18 treatment; Fine: 26.48% peNDF>1.18 treatment; Very fine: 24.75% peNDF>1.18 treatment.

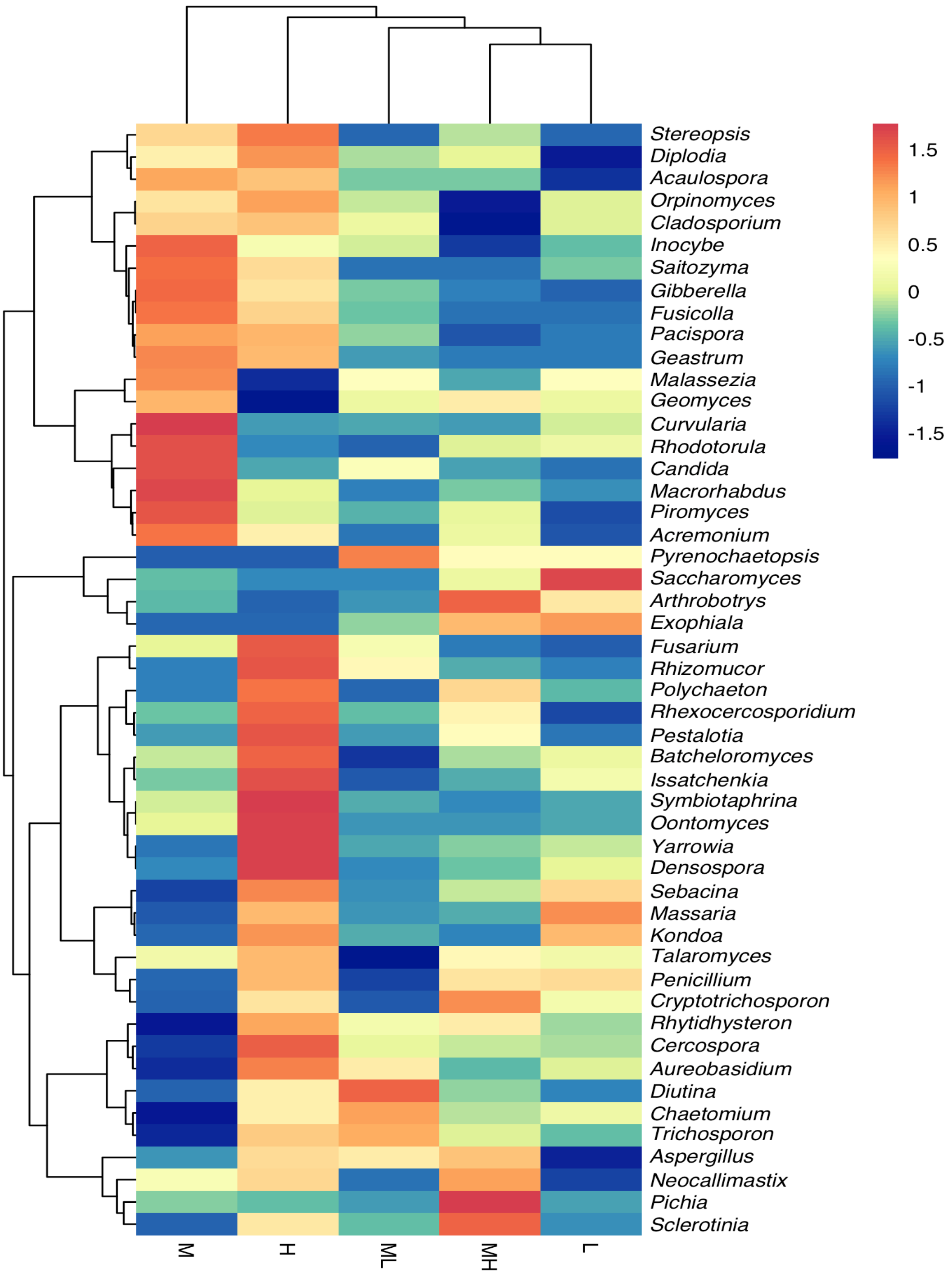

Figure S2. Heat map of species abundance at the genus level. Select the top 50 genus of abundance, cluster from the species and group, draw a heat map, each line is z-score standardized, the legend on the right sideshows z-score. The closer to red the color, the higher the relative abundance, while the closer to blue the color, the lower the relative abundance. Hierarchical clustering based on the distances of the five samples along the X-axis and the bacterial genera along the Y-axis are indicated in the upper part and on the left side of the figure, respectively. Long: 32.97% peNDF$_{>1.18}$ treatment; Medium: 29.93% peNDF$_{>1.18}$ treatment; Short: 28.14% peNDF$_{>1.18}$ treatment; Fine: 26.48% peNDF$_{>1.18}$ treatment; Very fine: 24.75% peNDF$_{>1.18}$ treatment.
